# Infectious agents and their physiological correlates in early marine Chinook salmon (*Oncorhynchus tshawytscha*)

**DOI:** 10.1093/conphys/coad031

**Published:** 2023-05-19

**Authors:** Yuwei Wang, Arthur L Bass, Scott G Hinch, Shaorong Li, Emiliano Di Cicco, Karia H Kaukinen, Hugh Ferguson, Tobi J Ming, David A Patterson, Kristina M Miller

**Affiliations:** Forest and Conservation Sciences, University of British Columbia, 3041-2424 Main Mall, Vancouver, BC, V6T 1Z4, Canada; Forest and Conservation Sciences, University of British Columbia, 3041-2424 Main Mall, Vancouver, BC, V6T 1Z4, Canada; Pacific Biological Station, Fisheries and Oceans Canada, 3190 Hammond Bay Rd, Nanaimo, BC, V9T 6N7, Canada; Forest and Conservation Sciences, University of British Columbia, 3041-2424 Main Mall, Vancouver, BC, V6T 1Z4, Canada; Pacific Biological Station, Fisheries and Oceans Canada, 3190 Hammond Bay Rd, Nanaimo, BC, V9T 6N7, Canada; Pacific Salmon Foundation, 1682 W 7th Ave, Vancouver, BC, V6J 4S6, Canada; Pacific Biological Station, Fisheries and Oceans Canada, 3190 Hammond Bay Rd, Nanaimo, BC, V9T 6N7, Canada; School of Veterinary Medicine, St. George’s University, University Centre Grenada, W. Indies, Grenada; Pacific Biological Station, Fisheries and Oceans Canada, 3190 Hammond Bay Rd, Nanaimo, BC, V9T 6N7, Canada; Fisheries and Oceans Canada, School of Resource and Environmental Mangement, Simon Fraser University, Science Branch, 643A Science Rd, Burnaby, BC, V5A 1S6, Canada; Forest and Conservation Sciences, University of British Columbia, 3041-2424 Main Mall, Vancouver, BC, V6T 1Z4, Canada; Pacific Biological Station, Fisheries and Oceans Canada, 3190 Hammond Bay Rd, Nanaimo, BC, V9T 6N7, Canada

## Abstract

The early marine life of Pacific salmon is believed to be a critical period limiting population-level survival. Recent evidence suggests that some infectious agents are associated with survival but linkages with underlying physiological mechanisms are lacking. While challenge studies can demonstrate cause and effect relationships between infection and pathological change or mortality, in some cases pathological change may only manifest in the presence of environmental stressors; thus, it is important to gain context from field observations. Herein, we examined physiological correlates with infectious agent loads in Chinook salmon during their first ocean year. We measured physiology at the molecular (gene expression), metabolic (plasma chemistry) and cellular (histopathology) levels. Of 46 assayed infectious agents, 27 were detected, including viruses, bacteria and parasites. This exploratory study identified:

- a strong molecular response to viral disease and pathological change consistent with jaundice/anemia associated with Piscine orthoreovirus,
- strong molecular signals of gill inflammation and immune response associated with gill agents `*Candidatus* Branchiomonas cysticola’ and *Parvicapsula pseudobranchicola,*
- a general downregulation of gill immune response associated with *Parvicapsula minibicornis* complementary to that of *P. pseudobranchicola*.

Importantly, our study provides the first evidence that the molecular activation of viral disease response and the lesions observed during the development of the PRV-related disease jaundice/anemia in farmed Chinook salmon are also observed in wild juvenile Chinook salmon.

## Teaser

Physiological parameters of early marine Chinook salmon, measured via gene expression and plasma chemistry, were correlated with the load of infectious agents confirmed in the host through *in situ* hybridization. Agents demonstrating the most associations with deviation from host homeostasis included Piscine orthoreovirus, *Candidatus* Branchiomonas cysticola, *Parvicapsula pseudobranchicola* and *Parvicapsula minibicornis.*

## Introduction

Infectious agents, including bacteria, parasites and viruses, are found in aquatic ecosystems worldwide, and impact both wild and cultured organisms. While diseased fish and associated mortalities are easily detected in an aquaculture setting, the impact of infectious diseases in wild fish populations is difficult to observe. Diseased organisms are more vulnerable to predation, making it difficult to sample individuals at a late stage of disease (Bakke and Harris, 1998; Miller *et al*., 2014; Furey *et al*., 2021). For this reason, and because fish can be easily accessed and a multitude of factors controlled in a closed system, much of our knowledge of the histopathological and physiological impacts of infectious agents comes from laboratory or aquaculture-based experiments. This is certainly the case for Pacific salmon, a group of species in which dozens of infectious agents have been documented.

Pacific salmon (*Oncorhynchus* spp.) are iconic species on the west coast of North America due to their ecological, cultural, economic and recreational importance. The productivity of Pacific salmon in the North Pacific Ocean is characterized by high interannual variability but steady declines in some species and populations have been evident for decades, especially towards the southern distribution of the genus (Riddell *et al*., 2013; Welch *et al*., 2021). In British Columbia, marine survival of Chinook salmon (*Oncorhynchus* tshawytscha) has declined over the past 20 years with smolt-to-adult returns for many populations now often falling below 1% (Welch *et al*., 2021). The Committee on the Status of Endangered Wildlife in Canada recently designated 12 of 26 southern British Columbia Chinook populations as endangered (COSEWIC, 2019). Although the reasons behind the decline in Chinook abundance are still in question, predation (Nelson *et al*., 2019), shifting marine conditions (Welch *et al*., 2021), global climate change (Crozier *et al*., 2021) and infectious disease (Miller *et al*., 2014) are suspected to contribute to the decline.

The early marine life history of Pacific salmon is a critical period where salmon are thought to have very low survival (Beamish and Mahnken, 2001; Cunningham *et al*., 2018; Claiborne *et al*., 2020). Infectious agents are considered to have sporadic impacts because of their ability to reproduce rapidly and to influence the host as population regulators and selective agents (Bakke and Harris, 1998). A recent study found that some infectious agents detected in fish during early marine residence were negatively associated with population-level survival and body condition for Chinook salmon (Bass *et al*., 2022). However, this study was correlative and included no assessment of disease state of individual fish. While providing evidence of causal relationships between pathogens and survival in wild populations is nearly impossible, physiological associations with agents could provide another layer of correlative evidence. These physiological associations could be established by utilizing well-established assessments, including blood metabolites and ions and histopathology, combined with modern molecular techniques.

The advancement of molecular technology has created more possibilities for studying infectious agents in wild populations. High-throughput quantitative polymerase chain reaction (HT-qPCR) enables rapid and highly sensitive screening of infectious agents across many samples (Miller *et al*., 2014, 2016). On the same HT-qPCR platform, host gene expression can be simultaneously profiled through the inclusion of assays targeting aspects of host health, such as immune status, stress level and osmoregulation (Jeffries *et al*., 2014; Teffer *et al*., 2017; Houde *et al*., 2019; Deeg *et al*., 2022). Such curated panels of genes can also be used to identify patterns indicating the host’s response to viral disease (Miller *et al*., 2017). Molecular technology, combined with traditional methods of studying fish physiology, such as blood chemistry and histopathology, is likely to broaden our understanding of infectious agents carried by wild fish and the relationship between infectious agents and early marine mortality.

In this study, we assessed associations between pathogen infection intensity and the physiology of wild juvenile Chinook salmon during their first year of marine residence. We compared infectious agent presence and loads to fish physiological conditions, including host transcription profiles, blood plasma metabolic and osmoregulatory variables (e.g. glucose, lactate, chloride, sodium and osmolality), and evidence of cellular damage through histopathology. We applied *in situ* analysis to co-localize specific infectious agents within the regions where cellular damage occurred to strengthen the observed relationships between infection and disease. We examined juvenile Chinook salmon from a broad geographic area, encompassing the waters surrounding Vancouver Island and the Salish Sea. This research is unique as we investigated associations between pathogens and three layers of physiological information: molecular (host gene expression), metabolic and ionic (blood plasma chemistry) and cellular (histopathology) of juvenile Chinook salmon in their early marine residence.

## Methods

### Methods overview

Juvenile Chinook salmon in their first marine year were collected by trawl along the British Columbia coast. Blood and multiple organs were collected from each fish. From blood samples, metabolic and ionic variables were assessed. A subsample of the organs was used for histopathological analysis including both hematoxylin and eosin staining and *in situ* hybridization. Six organs were pooled and screened for 46 pathogens by molecular analysis. Gill and liver tissue were separately prepared to measure the expression of host genes from several functional groups. For each pathogen, loads were compared to blood parameters and gene expression in linear mixed models that accounted for additional variation imposed by sampling season, location and life history. Model results were visualized in a heatmap so that pathogens with multiple associations across blood variables or gene groups, or those with particularly strong associations, could be identified.

### Fish, blood and tissue collection

**Figure 1:**
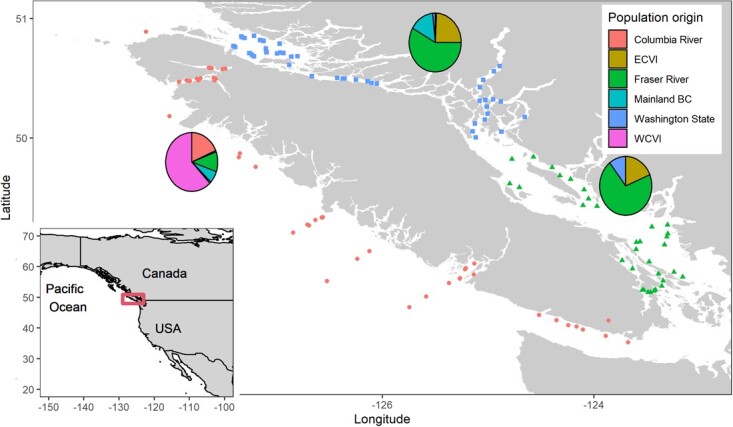
Capture locations (points) of Chinook salmon sampled around Vancouver Island (center) by mid-water trawl from 2012 to 2014. Red rectangle in inset map indicates the extent of the main map. Point color and shape represents the capture regions assigned for statistical analysis (red circles = West Coast Vancouver Island, green triangles = Southeast VI, blue squares = Northeast VI). For each capture region, pie charts depict the population origin for the sampled fish (colors in legend correspond to pie charts only; ECVI = East Coast Vancouver Island, WCVI = West Coast Vancouver Island).

Samples were obtained from Fisheries and Oceans Canada (DFO) research sampling programs along the southern coast of BC (Figure 1). All samples were obtained from the winter, summer and fall of 2012, 2013 and 2014. The sampling methods are described in Tucker *et al*. (2018). Briefly, fish were captured by midwater trawl (DFO marine sampling vessels) for 15 to 30 min at 5 knots and brought onboard. Juvenile Chinook salmon were haphazardly selected, and length (mm) and mass (g) were measured. Fish were dissected within 30 minutes of capture to optimize tissue quality for gene expression profiling. To ensure that only juvenile Chinook salmon were collected, seasonal size limits were applied as follows: May to August < 300 mm, October to November < 350 mm, February to March < 400 mm. Fin clip samples were collected from individuals using sterile scissors and preserved in 95% ethanol for genetic stock population identification (GSI) in the lab (Beacham *et al*., 2006). Whether an individual represented the subyearling or yearling life history at ocean entry was determined by the dominant life history in the natal population as determined by GSI.

Blood was collected from each individual using a sterile 26 gauge needle on a 1.0 mL syringe within 30 minutes of capture. Needles and syringes were flushed with heparin solution prior to blood extraction from the caudal peduncle. The collected blood samples were centrifuged at 6900 G for 5 minutes to isolate plasma for the measurement of physiological parameters in the laboratory, and then immediately stored at −80°C. Tissue biopsies were taken from the brain, kidney, liver and heart. Two separate sets of sterile dissection tools were used for exterior (gill) and interior tissues. Samples used for infectious agent detection and host gene expression profiling were collected first. Whole or half brain and tissue pieces of each of the other organs were individually preserved in vials of RNAlater (Qiagen, MD, USA), kept for 24 hours at 4°C and then frozen at −80°C or on dry ice. Additionally, tissue samples from gills, skeletal muscle, heart, liver, spleen, kidney, pancreas and brain (half) were also collected into histology cassettes and preserved in 10% buffered formalin. To maintain constant size of tissue samples, in the case of small specimens, the whole organ may be included (e.g. spleen); in the case of larger specimens, each tissue was then subsampled to a size not thicker than 4 mm in at least one of the three dimensions to allow formalin to penetrate and fix the tissues quickly.

### Molecular methods

Infectious agent detection and quantification and host gene transcriptome analyses were conducted at the Fisheries and Oceans Canada Pacific Biological Station (DFO-PBS, Nanaimo, BC, Canada). High-throughput quantitative PCR (HT-qPCR) using assays with TaqMan probes was run on the Fluidigm BioMark™ HT microfluidics platform (Fluidigm, CA, USA) to test for the presence and quantity of infectious agents and the expression of host genes. This technology has been adopted for salmon research (Miller *et al*., 2016) and used in several studies of Pacific salmon featuring both juvenile and adult fish (Jeffries *et al*., 2014; Teffer *et al*., 2017; Deeg *et al*., 2022). The platform performs independent PCR reactions for each of 96 samples against each of 96 assays for a total of 9216 reactions. The specificity, sensitivity and repeatability of the platform have been validated for use in salmon infectious agent detection and quantification (Miller *et al*., 2016).

BioMark™ dynamic arrays were run separately for infectious agents and host genes to maximize the number of agents and host genes surveyed. For infectious agent detection, 47 assays to 46 infectious agents (two assays to infectious salmon anemia virus) and one reference gene were selected to run in duplicate on each dynamic array. For infectious agent monitoring, each dynamic array contained combined DNA and cDNA from pooled samples from the brain, gill, kidney, liver and heart, positive and negative processing controls and six standard serial dilutions of combined artificial positive constructs (clones of DNA sequences corresponding to all infectious agent assays). Combining cDNA and DNA allows for detection of both RNA and DNA viruses, as well as detection of pathogens in both active and dormant states. For host gene expression, only a subset of gill samples (n = 218) and a subset of liver samples (n = 263) were available for analyses. cDNA from these single tissue samples was run separately on different dynamic arrays. At least one positive and one negative processing control and six standard serial dilutions (made by pooling host cDNA using 1 μL from every sample) were also allocated on every chip. Eighty-nine host gene assays were selected to run as singletons based on their known contributions to immune response, general stress response, osmolality, thermal and hypoxia stress, along with three reference genes (Table 1; Miller *et al*., 2011, 2016; Akbarzadeh *et al*., 2018). The selection included a set of genes identified previously as a “mortality-related signature” that has been predictive of wild adult salmon migration and spawning failure (Miller *et al*., 2011), as well as speed of migration into freshwater (Drenner *et al*., 2018). A panel of viral disease development (VDD) host genes was also included, which when co-expressed, can distinguish fish in a viral disease state from a carrier or no virus state (Miller *et al*., 2017). The VDD biomarker panel was developed using challenge studies and farm outbreak samples from multiple viral agents and salmon species. Not all assayed genes were included in subsequent analyses, including those which had poor assay efficiency and those that were part of a thermal stress panel which we decided was irrelevant for correlations with pathogen load (Table 1; sequences provided in Supplementary Material, Table S2).

Laboratory procedures for nucleic acid preparation and qPCR protocol are described in (Miller *et al*., 2014) and (Miller *et al*., 2016), and the same process has been applied in several recent studies (Jeffries *et al*., 2014; Teffer *et al*., 2017; Tucker *et al*., 2018; Deeg *et al*., 2022). In short, tissues from each sample were individually homogenized. For infectious agent detection, the aqueous phase of multiple tissues from the same fish was pooled to extract RNA and the organic layer pooled to extract DNA. For host gene profiling, aqueous samples for gill and liver tissue homogenates were used separately for RNA extraction. Extracted DNA and RNA were assessed for purity and normalized. cDNA was synthesized from normalized RNA, and for infectious agent monitoring, equal aliquots of cDNA and DNA were combined. cDNA alone was used for host transcriptome analyses. Because the individual wells on the microfluidics dynamic arrays for the BioMark platform are small volume (7 nL), samples must undergo an initial enrichment step involving amplification with all target assay primers combined according to the Fluidigm protocol (Miller *et al*., 2016). Prior to individual assay qPCR on the dynamic arrays, excess or unincorporated nucleotides and primers were removed, and samples were diluted 5-fold. Cycle threshold (Ct) was determined in the BioMark Real-Time PCR software. Amplification curves of all reactions between each assay and each sample were visually examined in case of any abnormal curve shape (in which case they could be manually scored as N/A or negative). Assay efficiencies were calculated based on a fitted curve from serial dilutions. Assays with efficiencies less than 80% or greater than 120% or coefficients of determination (R^2^) of the fitted curve less than 0.98 were removed from subsequent analyses. Host gene expression of gill and liver samples were normalized with the 2^−ΔΔCt^ method (Livak and Schmittgen, 2001) in which relative expression of each gene was calculated using the reference genes and the pooled sample made with all available same-tissue samples of the entire study included.

**Table 1:** Assays for host genes and infectious agents used in HT-qPCR analyses on juvenile Chinook salmon *(Oncorhynchus tshawytscha).* Functional group represents inclusion into specific biomarker panels in our analysis; note that while many genes can be involved in multiple physiological pathways, they were only included in a single functional group for our analysis. Mortality related signature (MRS) is a curated panel discovered in association with migratory losses in salmon (Miller *et al*., 2011) and contains genes from multiple physiological pathways. Viral disease development (VDD) is a curated panel that when co-expressed indicates a viral disease state across multiple RNA viruses infecting salmon (Miller *et al*., 2017). Sequences for all assays provided in Supplementary Material, Table S2.

**Symbol**	**Host gene/Infectious agent name**	**Assay Class**	**Functional group**
C1Qc	Complement C1q C Chain	Host gene	Adaptive immunity
CD4	cluster of differentiation 4	Host gene	Adaptive immunity
CD83	cluster of differentiation 83	Host gene	Adaptive immunity
CD8a	cluster of differentiation 8 subunit α	Host gene	Adaptive immunity
IgMs	Immunoglobulin	Host gene	Adaptive immunity
IgT	Immunoglobulin tau	Host gene	Adaptive immunity
IL-15	Interleukin 15	Host gene	Adaptive immunity
MHC1	Major histone compatibility complex 1	Host gene	Adaptive immunity
MHCII-B	Major histone compatibility complex class II	Host gene	Adaptive immunity
TCRa	T-cell receptor alpha	Host gene	Adaptive immunity
TCRb	T-cell receptor beta	Host gene	Adaptive immunity
TNF	Tumor necrosis factor	Host gene	Adaptive immunity
C3	Complement factor 3	Host gene	Innate immunity
hep	Hepcidin	Host gene	Innate immunity
IL-11	Interleukin 11	Host gene	Innate immunity
IL-17D	Interleukin 17D	Host gene	Innate immunity
IL-1B	Interleukin 1b	Host gene	Innate immunity
IL-8	Interleukin 8	Host gene	Innate immunity
MMP13	Matrix metalloproteinase 13	Host gene	Innate immunity
MMP25	Matrix metalloproteinase 25	Host gene	Innate immunity
PCBL	Precerebellin	Host gene	Innate immunity
SAA	Serum amyloid protein a	Host gene	Innate immunity
TF	transferrin	Host gene	Innate immunity
ALDOA	Aldolase A	Host gene	Metabolism
COX6B1	Cytochrome c oxidase subunit 6B1	Host gene	Metabolism
glut2	Glucose transporter 2	Host gene	Metabolism
IDH3B	Isocitrate Dehydrogenase 3 Beta	Host gene	Metabolism
Ldhb	L-lactate dehydrogenase B-A chain-like	Host gene	Metabolism
MPDU1	Mannose-P-Dolichol Utilization Defect 1	Host gene	Metabolism
PgK3	Phosphoglycerate kinase 3	Host gene	Metabolism
COMMD7	COMM domain-containing protein 7	Host gene	MRS
FYB	FYN-T-binding protein	Host gene	MRS
HTA	HIV-1 Tat interactive protein	Host gene	MRS
IRF1	Interferon regulatory factor 1	Host gene	MRS
JUN	Transcription factor	Host gene	MRS
KRT8	Cyclokeratin-8	Host gene	MRS
PRAS	G-protein mRNA	Host gene	MRS
RPL31	60S Ribosomal protein L31	Host gene	MRS
SCG	secretogranin II [Ctenopharyngodon idella]	Host gene	MRS
STAT1	Signal transducer and activator of transcription 1-alpha/beta	Host gene	MRS
CA4	Carbonic anhydrase 4	Host gene	Osmoregulation
CCL4	Chemokine (C-C motif) ligand 4	Host gene	Osmoregulation
CFTR-I	Cystic fibrosis transmembrane conductance regulator I	Host gene	Osmoregulation
EF-2	Elongation factor 2	Host gene	Osmoregulation
HBA	Hemoglobin subunit α	Host gene	Osmoregulation
NKA_a3	Na+/K+ ATPase subunit a3	Host gene	Osmoregulation
NKA_b1	Na+/K+ ATPase subunit b1	Host gene	Osmoregulation
NKAa1-b	Na+/K+ ATPase subunit α-1b	Host gene	Osmoregulation
NKAA1C	Na+/K+ ATPase subunit 1c	Host gene	Osmoregulation
park7	Protein deglycase DJ-1	Host gene	Osmoregulation
52Ro	52 kDa Ro protein-2	Host gene	VDD
CA054694	Mitochondrial ribosomal protein (VAR1)	Host gene	VDD
CD9	cluster of differentiation 9	Host gene	VDD
DEXH	ATP-dependent RNA helicase	Host gene	VDD
GAL3	Galectin-3-binding protein precursor	Host gene	VDD
HERC6	Probable E3 ubiquitin-protein ligase HERC6	Host gene	VDD
IFI44a	Interferon-induced protein 44 alpha	Host gene	VDD
IFIT5	Interferon-induced protein with tetratricopeptide repeats 5	Host gene	VDD
IFNa	Interferon alpha	Host gene	VDD
Mx_onts	Antiviral protein	Host gene	VDD
NFX	Zinc finger NFX1-type	Host gene	VDD
RSAD	Radical S-adenosyl methionine domain-containing protein 2	Host gene	VDD
SRK2	Tyrosine-protein kinase SKR2	Host gene	VDD
VHSV-P10	VHSV-induced protein-10 mRNA	Host gene	VDD
78d16.1	S100 calcium binding protein	Reference Gene	Reference gene
COIL-p84	Coiled-coil domain-containing protein 84	Reference Gene	Reference gene
MrpL40	39S ribosomal protein L40	Reference Gene	Reference gene
ae_hyd	Aeromonas hydrophila	Infectious agent	Bacteria
ae_sal	*Aeromonas salmonicida*	Infectious agent	Bacteria
c_b_cys	*‘Candidatus* Branchiomonas cysticola’	Infectious agent	Bacteria
fl_psy	*Flavobacterium psychrophilum*	Infectious agent	Bacteria
sch	*Candidatus* Syngnamydia salmonis	Infectious agent	Bacteria
mo_vis	*Moritella viscosa*	Infectious agent	Bacteria
pch_sal	*Piscichlamydia salmonis*	Infectious agent	Bacteria
pisck_sal	*Piscirickettsia salmonis*	Infectious agent	Bacteria
re_sal	*Renibacterium salmoninarum*	Infectious agent	Bacteria
rlo	Rickettsia-like organism	Infectious agent	Bacteria
te_mar	*Tenacibaculum maritimum*	Infectious agent	Bacteria
vi_ang	*Vibrio anguillarum*	Infectious agent	Bacteria
vi_sal	*Vibrio salmonicida*	Infectious agent	Bacteria
ye_ruc	*Yersinia ruckeri*	Infectious agent	Bacteria
de_sal	*Dermocystidium salmonis*	Infectious agent	Mesomycetozoean
ic_hof	*Ichthyophonus hoferi*	Infectious agent	Mesomycetozoean
sp_des	*Sphaerothecum destructuens*	Infectious agent	Mesomycetozoean
fa_mar	*Facilispora margolisi*	Infectious agent	Microsporidium
lo_sal	*Loma salmonae*	Infectious agent	Microsporidium
nu_sal	*Nucleospora salmonis*	Infectious agent	Microsporidium
pa_ther	*Paranucleospora theridion*	Infectious agent	Microsporidium
ce_sha	*Ceratonova shasta*	Infectious agent	Myxozoan
ku_thy	*Kudoa thyrsites*	Infectious agent	Myxozoan
my_arc	*Myxobolus arcticus*	Infectious agent	Myxozoan
my_ins	*Myxobolus insidiosus*	Infectious agent	Myxozoan
pa_kab	*Parvicapsula kabatai*	Infectious agent	Myxozoan
pa_min	*Parvicapsula minibicornis*	Infectious agent	Myxozoan
pa_pse	*Parvicapsula pseudobranchicola*	Infectious agent	Myxozoan
te_bry	*Tetracapsuloides bryosalmonae*	Infectious agent	Myxozoan
gy_sal	*Gyrodactylus salaris*	Infectious agent	Platyhelminthes
na_sal	*Nanophyetus salmincola*	Infectious agent	Platyhelminthes
cr_sal	*Cryptobia salmositica*	Infectious agent	Protozoan
ic_mul	*Ichthyophthirius multifiliis*	Infectious agent	Protozoan
ne_per	*Neoparamoeba perurans*	Infectious agent	Protozoan
sp_sal	*Spironucleus salmonicida*	Infectious agent	Protozoan
ihnv	Infectious hematopoietic necrosis virus	Infectious agent	Virus
ipnv	Infectious pancreatic necrosis virus	Infectious agent	Virus
isav7	Infectious salmon anemia virus	Infectious agent	Virus
isav8	Infectious salmon anemia virus	Infectious agent	Virus
pspv	Pacific salmon parvovirus	Infectious agent	Virus
pmcv	Piscine myocarditis virus	Infectious agent	Virus
prv	Piscine orthoreovirus	Infectious agent	Virus
sav	Salmon alphavirus 1, 2, and 3	Infectious agent	Virus
omv	Salmonid herpesvirus	Infectious agent	Virus
ver	Viral encephalopathy and retinopathy virus	Infectious agent	Virus
ven	Viral erythrocytic necrosis virus	Infectious agent	Virus
vhsv	Virus Viral hemorrhagic septicemia virus	Infectious agent	Virus

### DNA natal population identification

Fin clip samples preserved in ethanol were used to assess the population of origin for individual fish following methods outlined in Beacham *et al*. (2006). Stock IDs were grouped into six geographical natal groups: West Coast of Vancouver Island, East Coast of Vancouver Island, Fraser River system (upper and lower Fraser River and Thompson River), Mainland BC (including streams in Northern, Central and Southern mainland BC that were not included in the other five region groups), the Columbia River system (including Columbia River and Snake River) and Washington (including tributaries to the Puget Sound and Strait of Juan de Fuca). Hereafter, they will be referred to as WCVI, ECVI, Fraser, Mainland, Columbia and Washington, respectively. In the analysis, individuals from an unknown natal population or with a probability of assigned natal population less than 0.50 were excluded.

### Blood analyses

Blood samples were collected for a subset of juvenile Chinook salmon (n = 213) to examine plasma concentrations of lactate, glucose, chloride, sodium osmolality, using methods identical to Teffer *et al*. (2017). The analyses were conducted at the DFO West Vancouver Laboratory, BC. Immediately after thawing the 1500 μL centrifuge tube, the plasma layer inside the tube was carefully transferred into a new 500 μL centrifuge tube by a single-use pipet. Plasma glucose and lactate concentrations were measured using a YSI 2300 Stat Plus lactate/glucose analyzer (Yellow Springs Instruments, OH, USA). Chloride concentration and osmolality were measured as the average of the duplicates using a Model 4 425 000 Haake Buchler digital chloridometer and the Advanced Instruments 3320 freezing point osmometer, respectively. If the disagreement between the duplicates was greater than 3 mmol/L for chloride or 3 mOsm/kg for osmolality, measurements were repeated and the average was taken from the two closest measurements. At a later date, transferred plasma samples were thawed again and were diluted at 1:100 dilution for sodium analysis by a BWB XP flame photometer. The photometer was calibrated against a four-point standard curve that was created using sodium standard solutions at every start-up or after a change was observed during checks performed every ten samples. If the difference between the two results was greater than 6 mmol/L, the measurements were repeated and the averages were calculated using the two closest results. Because the capture process causes rapid changes to most of these plasma variables (Pickering *et al*., 1982) and blood was collected anywhere from 15 to 60 minutes following the initiation of a trawl tow, the measures we collected should be considered representative of fish experiencing a capture stressor.

### Histology

Based on the work of Tucker *et al*. (2018), findings herein and other observations during the course of the research conducted under the Strategic Salmon Health Initiative, *Ceratonova shasta, Parvicapsula minibicornis, Paranucleospora theridion*, PRV, ‘*Candidatus* Branchiomonas cysticola’ and *Ichthyophonus hoferi* were chosen for histological analysis. Forty-four histology samples that tested PCR-positive (generally with loads exceeding 1000 copies per μg tissue) for at least one of these six infectious agents were used (Supplementary Material, Table S3). Samples were dehydrated through an ascending gradient of ethanol solutions. Samples were then embedded in paraffin wax, and consecutive serial sections were cut at 3.5 μm thickness. One section per sample was stained with standard hematoxylin and eosin (H&E) for histological examination. To localize the target agent in the host tissues, thus providing further evidence of its association with cellular damage, 28 samples with relatively high loads of five infectious agents (*C. shasta, Parvicapsula minibicornis, ‘Ca.* B. cysticola’*, I. hoferi* and PRV) or showing lesions in tissues consistent with infections from these agents were used for *in situ* hybridization (ISH) staining, which used probes designed to hybridize to specific infectious agent RNA. The ISH was implemented using BASEscope™ Assay RED (for PRV), RNAscope® 2.5 HD Duplex Assay (for *C. Shasta* and *Parvicapsula minibicornis*) and RNAscope® 2.5 HD RED (for *‘Ca*. B. cysticola’ and *I. hoferi)* (Advanced Cell Diagnostics, Newark, CA, USA) according to the instructions from the manufacturer. In preparation for hybridization, consecutive serial dewaxed sections to the ones used for the histopathological analysis were boiled for 30 minutes in RNAscope target retrieval reagents (Advanced Cell Diagnostics, Newark, CA, USA) and then incubated for 30 minutes either in RNAscope Protease III (PRV) or Protease Plus (*C. Shasta* and *Parvicapsula minibicornis, ‘Ca.* B. cysticola’ and *I. hoferi*) reagent prior to hybridization. The sections were then hybridized with a BASEscope (PRV), RNAscope Duplex (*C. Shasta* and *Parvicapsula minibicornis*) or RNAscope® 2.5 HD (*‘Ca.* B. cysticola’ and *I. hoferi*) probes against a portion of target agent genome segment (Advanced Cell Diagnostics, Newark, California, catalog #705151[PRV], #512401 [*C. Shasta*], #512381 [*Parvicapsula minibicornis*], #823971 [‘*Ca.* B. cysticola’] and #823981 [*I. hoferi*]), to detect the target agent in the tissues. Probes against the bacterial gene dapB were used as a negative control to confirm the absence of background and/or of non-specific cross-reactivity of the assay (Advanced Cell Diagnostics, catalog #701021 [for BASEscope protocol] and #310043 [for both RNAscope protocols]). Two samples resulted negative through qPCR to all the agents used for the ISH were also utilized as negative controls to confirm the absence of cross-reactivity. A probe against the housekeeping gene PPIB (Advanced Cell Diagnostic, catalog #540651) was used to assess the quality of RNA present in the tissue sections. The finished histopathological sections went through the first round of visual exam by a veterinary pathologist (co-author EDC), and then were read by a second fish pathologist (co-author HF). Lesions were scored as mild, medium or severe (i.e. 1 to 3, respectively). All images captured from the slides were photographed by a camera system (Nikon Digital Sight DS-U3, Nikon, ON, Canada) attached to the Nikon Eclipse Ni microscope (Nikon, ON, Canada) and generated by Nikon NIS-Elements D4.30.01 64 Mb software.

### Statistical analyses

All statistical analyses were performed in R statistical software, version 3.4.2 (R Core Team, 2017). Infectious agent load was defined as the amount of infectious agent nucleic acids in a given sample. The infectious agent Ct values were first averaged between replicates. In the case where an infectious agent was not positive for both replicates, no detection was assigned. The infectious agent Ct values were then converted to copy numbers using the standard curve method (Larionov *et al*., 2005). For this study, we defined limit of detection (LOD) as a cycle threshold (Ct) number below which true positive results were expected 75% of the time for a given assay, based on reanalysis (unpublished) of Miller *et al*. (2016) data that included multiple independent serial dilutions. Due to the high sensitivity of the BioMark platform, this is a conservative estimate. Data exceeding the LOD for a given pathogen were replaced with zero.

For each individual, we calculated the total number of infectious agent taxa detected (total pathogen taxa). Because this metric does not take the load of infectious agents per individual into account, Relative Infectious Burden (RIB) (Bass *et al*., 2019) was also calculated. This metric is a composite score that divides the load of each agent detected in an individual by the highest load of that agent in the population and then sums the values for the individual across agents. For each infectious agent, prevalence was calculated as the percentage of positive detections within the entire study population.

To test for correlations between infectious agents (plus RIB and total pathogen taxa) and blood plasma variables, we employed linear mixed models individually for each pathogen and blood plasma variable combination (100 models). For a given model, the blood plasma variable was the response, pathogen load was the explanatory variable of interest and smolt age (subyearling vs yearling), season and capture region were included to account for variation in the plasma variable unrelated to pathogen load. Season and capture region were random intercepts. Capture regions were West Coast Vancouver Island, Northeast Vancouver Island and Southeast Vancouver Island (Figure 1). The plasma variable and pathogen load were both scaled by one standard deviation so that associations could be visually compared across pathogens and associations could be discussed in terms of standard deviation changes in both predictor and response. Pathogen load was transformed by adding 1 and log-transforming prior to scaling. This analysis and similar models for gene expression were only conducted for infectious agents with 1% prevalence or greater (18 pathogens), excluding *Nanophyetus salmincola*, which happened to have an insufficient number of positive fish for which samples were run for plasma and gene expression. Note that all models were also applied to RIB and total pathogen taxa.

**Figure 2:**
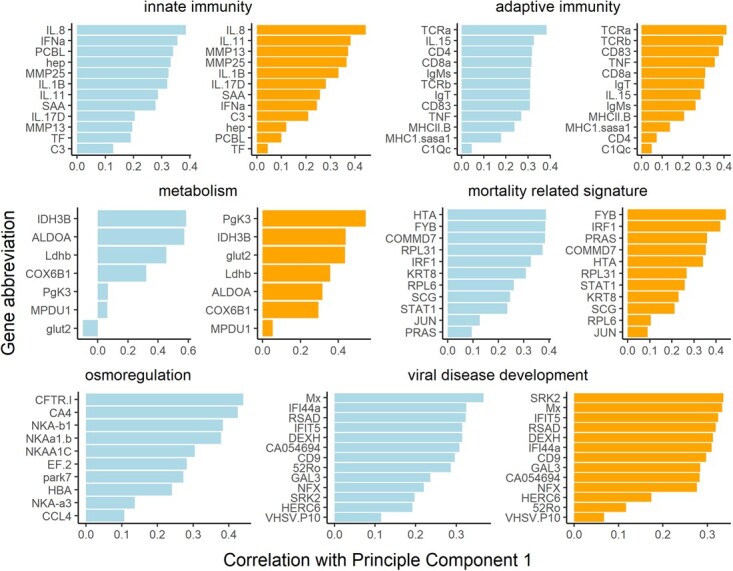
Correlations of gene expression (2^−ΔΔCt^) for individual genes with principal component 1 for each functional gene group. Gill is presented on left (blue) and liver is presented on right (orange). Osmoregulation gene expression was only analyzed for gill tissue.

A similar approach was used to test for correlations between infectious agent load and fish physiology at the molecular level in both gill and liver tissue. The response variable for gene expression was the first principal component (PC1) from separate principal component analyses for each of six functional gene groups (Table 1, Figure 2), resulting in 120 models per tissue (18 pathogens and 2 coinfection variables modeled against 6 functional gene groups). Most of these functional groups were derived from previously developed, curated biomarker panels (mortality-related signature; Miller *et al*., 2011); osmoregulation (Houde *et al*. 2019a; Akbarzadeh *et al*., 2018) and viral disease development (Miller *et al*., 2017). For most curated panels, the presence of a given physiological state is predicted by the co-upregulation of most or all of the genes on the panel. The remaining three groups were innate immune response, adaptive immune response and metabolism. Note that many genes can have roles in more than one physiological pathway/group, but in our analysis, they were only represented once. Separate PCAs were conducted for each tissue and the PC1 axis was flipped where necessary so that PC1 had similar gene loadings between gill and liver tissue for each functional group (Figure 2). The primary difference in model structure from the plasma analysis was that dynamic array ID number (Biomark PCR chip) was included as a random intercept to account for variance due to analytical (laboratory) artifacts. Because samples were non-randomly distributed across dynamic arrays, capture region was confounded by dynamic array ID and thus could not be included as a random intercept. For confirmation in the interpretation of associations between pathogens and PCs, the same model structure was applied to each gene in gill and liver individually (1560 models for gill, 1520 for liver; heatmaps provided in Supplementary Material, Fig. S5, S6).

Coefficients for pathogen loads were presented for plasma variables and gene expression in a heatmap using the pheatmap() function from the R package pheatmap (Kolde and Kolde, 2015). We allowed the pheatmap() function to cluster both pathogens and response variables (plasma variables and gene expression) using the default “complete linkage” clustering method. To reduce Type I errors, we adjusted all the *p* values using the false discovery rate (FDR) approach across all 323 model results composing the heatmap (Benjamini and Hochberg, 1995). For inference we prioritized pathogens with correlations that were significant following FDR *P* value adjustment and showed consistent patterns across response variables. However, pathogens with multiple *P* values < 0.05 prior to FDR adjustment are also described.

## Results

**Figure 3:**
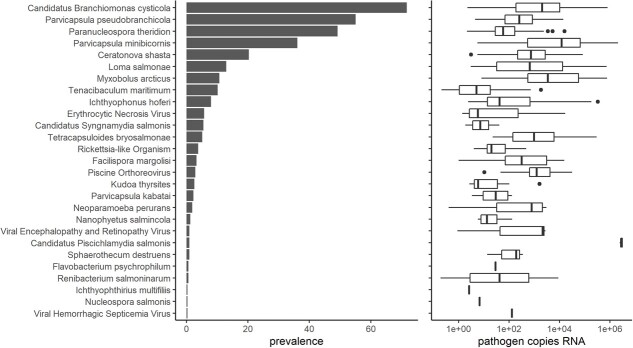
Prevalence and load of all pathogens detected in Chinook salmon sampled around Vancouver Island, 2012–2014. Load data are presented on the log10 scale (only positive detections included in boxplot).

Twenty-seven of 46 assayed infectious agent taxa were detected at least once within the limit of detection among 319 juvenile Chinook salmon (Figure 3). Nineteen infectious agents had an overall prevalence greater than 1%, including two viruses, four bacteria and 13 parasites (Figure 3). ‘*Ca.* Branchiomonas cysticola’ was the most prevalent and was found in 71.8% of the total sample, whereas viral hemorrhagic septicemia virus (VHSV) was the least prevalent, only detected in one individual (Figure 3). Across the entire study population, total pathogen taxa ranged from 0 to 7 pathogens per individual with a median of 3 and a mean of 2.97 (± SD, 1.37).

Glucose was the only plasma variable that did not appear noticeably elevated above the baseline estimates we drew from the literature (Supplementary Material, Table S1). At an overall mean 3.3 ± 1.2 (SD) mmol/L, plasma glucose in our study appeared generally lower than literature values which ranged from 4 to 6 mmol/L (Supplementary Material, Table S1). Plasma ions were all moderately elevated (Supplementary Material, Table S1) in trawl collected Chinook; chloride averaged 154.6 ± 17.3 mmol/L (baseline = 128–140 mmol/L), sodium averaged 171.2 ± 15.4 mmol/L (baseline = 152–170 mmol/L) and osmolality averaged 373.4 ± 33.5 mOsm/kg (baseline = 332–372 mOsm/kg). At an average of 13.4 ± 3.3 mmol/L, lactate was considerably higher for trawl caught Chinook than the baseline 2.2 to 3.6 mmol/L, including for troll caught fish sample 1 to 1.5 hours after capture (8.3 ± 2.5 mmol/L; Parker *et al*. 1959).

### Model results

**Figure 4:**
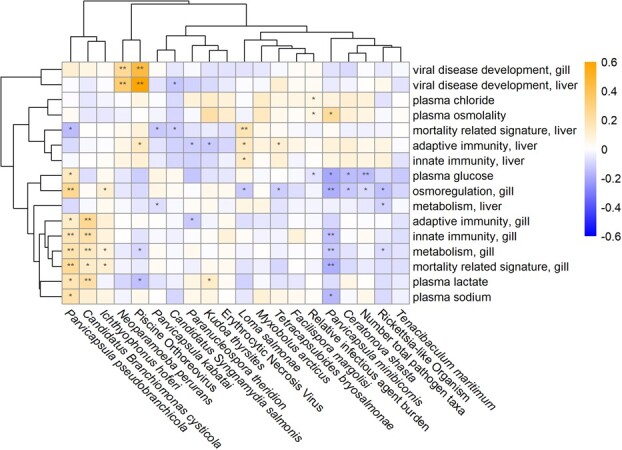
Heatmap of coefficients for each pathogen in models for blood plasma variables and the first principal components of functional gene groups in gill and liver tissue. Cell values represent the change in units of standard deviation for the plasma or gene variable associated with a standard deviation increase in log pathogen load (from mixed tissue). Two asterisks in a cell indicate an FDR-adjusted *p* value < 0.05 and a single asterisk indicates a *p* value < 0.05 prior to FDR adjustment.

Eighteen pathogens, total pathogen number and RIB were included as the explanatory variable of interest in mixed models with plasma variables, PC1s for each gill gene group and PC1s for each liver gene group as the response variables. On the y-axis of the heatmap (Figure 4), physiological responses sometimes clustered in logical ways (e.g. plasma osmolality and chloride, immune gene groups) and for gene expression, often within tissues (i.e. liver and gill gene groups largely grouped separately, Figure 4). Viral disease development was the only gene group to cluster across tissues. Plasma glucose and gill osmoregulation genes clustered closely with many similarities across pathogens (Figure 4). This clustering arose partly from a high number of negative associations for each of these physiological parameters. Osmoregulation in gill tissue, for example, was negatively associated with pathogen load for five pathogens and total pathogen taxa prior to FDR adjustment (Figure 4). In both tissues, mortality-related signature genes clustered among the immune gene groups (Figure 4).

**Figure 5:**
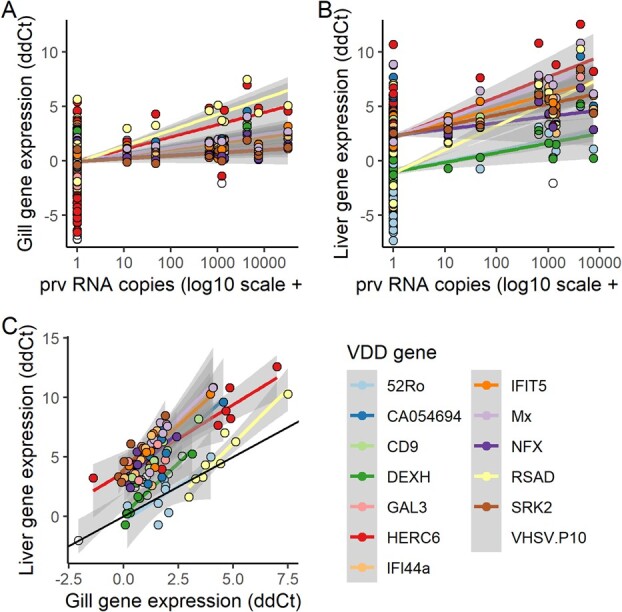
The expression of genes associated with viral disease development (VDD) showed a positive correlation with piscine orthoreovirus load in gill (A, 9 positives in 292 samples) and liver (B, 9 positives in 263 samples) tissues of Chinook salmon captured during their first ocean year. Furthermore, VDD gene expression in gill and liver were well-correlated across individuals with PRV detections (C). Lines indicate a simple linear regression for each gene with accompanying 95% confidence interval (gray fill). Black line in C represents a 1:1 relationship.

For gene groupings, associations with pathogen load tended to be exclusive to gene expression in one tissue or another but not both, with the exception being the VDD genes and Piscine orthoreovirus (PRV; Figures 4, 5). Note that correlations between *Neoparamoeba perurans* and VDD genes appear very similar to those for PRV, but are reflective of co-infections with PRV (described below). `*Candidatus* Branchiomonas cysticola’ and *Parvicapsula pseudobranchicola* demonstrated positive associations between gene expression and pathogen load across multiple gene groups in gill tissue but neutral associations in liver tissue (Figures 4, Supplementary Material, Fig. S5). *Parvicapsula minibicornis* had negative associations between gene expression and pathogen load in gill tissue but no strong patterns in liver tissue. *Loma salmonae* showed positive associations between liver gene expression and pathogen load and neutral expression in gill tissue (Figures 4, Supplementary Material, Fig. S5). Finally, `*Candidatus* Syngnamydia salmonis’ showed negative associations in liver tissue and neutral expression in gills (Figures 4, Supplementary Material, Fig. S5).

### Plasma variables and gene expression in select pathogens

#### Piscine orthoreovirus

The first and second strongest associations between pathogen load and a response variable in the study were between PRV load and the viral disease development PC1 (VDD-PC1) in liver (β = 0.60, *T*_247.4_ *=* 12.2, *p <* 0.001, n = 263) and gill (β = 0.44, *T*_281.4_ *=* 9.4, *p <* 0.001, n = 292) tissue (Figures 4, 5). Viral disease development gene expression was well correlated between gill and liver tissue for PRV-infected fish (Figure 5C). Prior to FDR adjustment, PRV was also negatively associated with plasma lactate and gill metabolism and positively associated with adaptive immunity genes in liver tissue (Figures 4, Supplementary Material, Fig. S6).

While it appears that *N. perurans* load was associated with VDD-PC1 (Figure 4), this pattern was an artifact of the data. All of the six fish with positive detections of *N. perurans* were captured in Quatsino Sound on March 10 and 11, 2013 and were from the Marble River population. Four of these six fish tested positive for PRV (there were a total of nine PRV-positive fish in the study), with three of those with PRV copy numbers exceeding 1000. It is highly unlikely that *N. perurans*, an extracellular parasite, would trigger activation of VDD genes, which show no activation in the presence of extracellular bacteria or a parasite (Miller *et al*., 2017).

#### *`Candidatus* Branchiomonas cysticola’

‘*Ca.* B. cysticola’ was positively associated with PC1 for metabolism, adaptive immunity and innate immunity in gill tissue (Figure 4). Plasma lactate was also positively associated with ‘*Ca.* B. cysticola’ load. Prior to FDR adjustment, this bacterium was positively associated with mortality-related signature expression in gill tissue.

#### Parvicapsula pseudobranchicola

This marine-transmitted myxozoan had positive associations with gill gene expression in all the functional groups assayed excluding viral disease development. Following FDR adjustment, osmoregulation, metabolism, innate immunity and mortality-related signature were all significantly associated (Figure 4). Of the plasma variables, lactate, glucose and sodium were positively associated with *P. pseudobranchicola* prior to FDR adjustment. *Parvicapsula pseudobranchicola* and ‘*Ca.* B. cysticola’, both known gill pathogens, presented similar patterns in gill gene expression, which led to their clustering in Figure 4.

#### Parvicapsula minibicornis

Another *Parvicapsula* species, *P. minibicornis*, had a pattern of associations with plasma variables and gill gene expression that was complementary to the pattern of *P. pseudobranchicola* (Figure 4). There was a negative association across gill gene expression groups and metabolism, osmoregulation, innate immunity and mortality-related signature following FDR adjustment. Plasma glucose and sodium were negatively associated with *P. minibicornis* load and plasma osmolality was positively associated, all prior to FDR adjustment.

#### Loma salmonae

This marine microsporidian was positively associated with gene groups in liver tissue. Mortality-related signature genes were significantly positively associated following FDR adjustment and immune cell signaling and adaptive and innate immunity genes were positively associated prior to adjustment.

#### Number of pathogen taxa and relative infectious burden

Number of pathogen taxa (per individual) was negatively associated with plasma glucose and osmoregulatory gene expression in gill prior to FDR adjustment (Figure 4). Relative infectious burden was positively associated with plasma chloride and osmolality and negatively associated with plasma glucose prior to FDR adjustment (Figure 4).

#### Histology

Lesions were observed on host tissues consistent with damage and disease development associated with five of the six agents of interest (*C. shasta, P. minibicornis,* PRV, *‘Ca.* B. cysticola’ and *I. hoferi*, but not *P. theridion*). The majority of lesions associated with these agents were on spleen and kidney, tissues we did not examine for gene expression (Supplementary Material, Table S3). Associations between lesions and the suspected causal agents were further supported by localization of the target agents near and/or in the lesions after applying ISH on the set of consecutive sections to those used for H&E staining (Supplementary Material, Table S3). No lesions consistent with disease development stemming from infection by *Paranucleospora theridion* were observed among the four individuals examined. Noteworthy findings of lesions consistent with each agent are described below with additional images provided in Supplementary Material, Figs. S1--S4.

#### Piscine orthoreovirus

**Figure 6:**
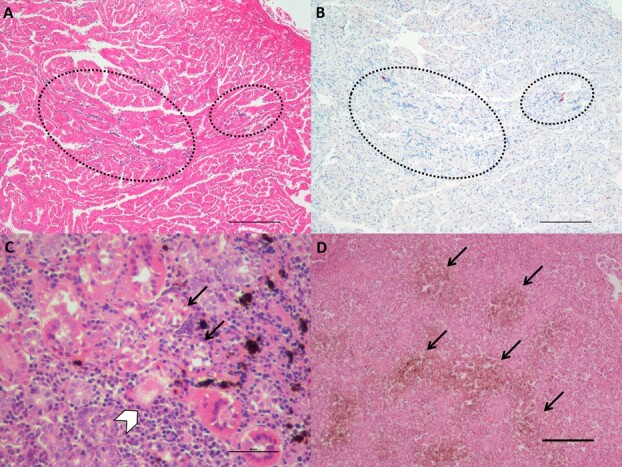
Piscine orthoreovirus positive (PRV+) fish had lesions consistent with PRV-related jaundice/anemia (Fish B2159). A) Hematoxylin and eosin (H & E) staining shows focal endocarditis (dotted circles) in the spongy layer of the myocardium (scale bar = 200 μm). B) *In situ* hybridization of the same field reveals PRV in focal inflammatory infiltrates. C) Renal tubular vacuolar degeneration (arrows) leading to tubule necrosis (white arrowhead) in the kidney (scale bar = 50 μm). D) Blood congestion and hemosiderin deposits (arrows) in the spleen (scale bar = 1000 μm).

Six of the eight fish examined contained at least one lesion consistent with PRV-related jaundice/anemia, with one containing mild, focal endocarditis in the spongy layer of the myocardium (Figure 6A, Supplementary Material, Table S3), one with renal tubular vacuolar degeneration leading to necrosis of kidney tubules (Figure 6C); two with hemosiderin (excess of hemoglobin byproduct indicative of red blood cell [RBC] lysis) and mild congestion and hyperplasia of the white pulp in the spleen (Figure 6D) and six with mild congestion and hyperplasia of the hemopoietic tissue in the kidney. One fish also presented with inflammation in the white skeletal muscle. Overall, inflammation in the spleen and hyperplasia of the hemopoietic tissue in the kidney was noted, along with some cases of mild necrosis in liver, spleen ellipsoids and renal tubules.

Using ISH, PRV was found in the host heart, posterior kidney, spleen, intestine and liver. In the heart, PRV was present in the cardiomyocytes involved in the focal inflammatory lesions observed in the spongy layer of the ventricle (Figure 6B). PRV was widely distributed in the red blood cells and macrophages of the spleen, where blood congestion and hemosiderin deposits were also observed (Supplementary Material, Fig. S1B). Red blood cells and macrophages in the posterior kidney were also heavily infected by the virus, and PRV was observed in association with a few necrotic renal tubules (Supplementary Material, Fig. S1A). In the intestine and liver, PRV was also found in the enterocytes and hepatocytes, respectively (Supplementary Material, Fig S1C and S1D).

#### *`Candidatus* Branchiomonas cysticola’

**Figure 7:**
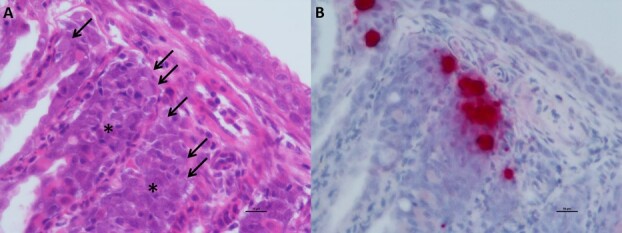
Epitheliocysts in the gill lamellae contained ‘Candidatus Branchiomonas cysticola’ (Fish B6930). A) Epitheliocysts in the gill lamellae (arrows) and associated proliferative/inflammatory reaction (asterisks, scale bar = 10 μm). B) Same field, using ISH to confirm that the epitheliocysts were infected with ‘Ca. B. cysticola’ (in red, scale bar = 10 μm).

Two of seven samples tested showed the presence of epitheliocystis in the gills’ secondary lamellae (Figure 7A), but only one of them also showed mild inflammation in the gills. By ISH, ‘*C.* B. cysticola’ was found inside the epitheliocystis observed in the gills, with some clusters of the bacteria where the cysts were developing (Figure 7B).

#### Parvicapsula minibicornis

**Figure 8:**
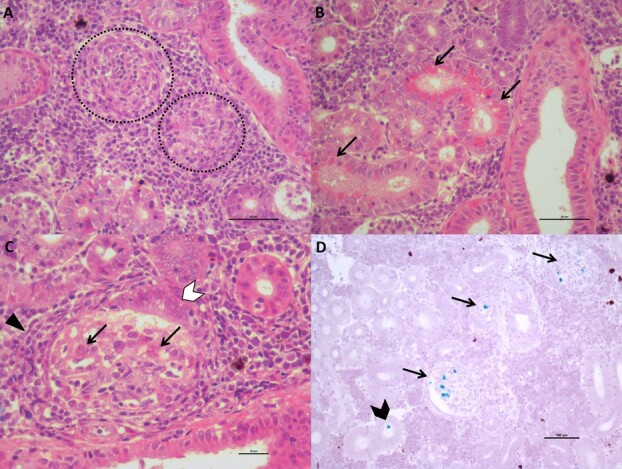
Lesions consistent with *Parvicapsula minibicornis*, and the pathogen itself, were found in kidney tissues (Fish B5083). A) Two different degrees of Glomerulonephritis (dashed circle): the glomerulus on the right is in a more advanced stage of necrosis (moderate), while the one on the left still shows a few morphological features (mild) and generalized interstitial hyperplasia (scale bar = 50 μm) B) Eosinophilic lipoprotein droplets (arrows) were visible in some renal tubules (scale bar = 50 μm). C) Glomerulonephritis (triangle head), hypertrophy/hyperplasia of Bowman’s capsule (white arrowhead) and *P. minibicornis* pre-sporogonic forms (arrows) (scale bar = 20 μm). D) *P. minibicornis* (blue) detection by ISH on both glomeruli (arrows) and in the lumen of renal tubules (black arrowhead) (scale bar = 100 μm).

Two of 14 fish examined had mild to moderate glomerulonephritis with mild necrosis of the renal tubules (Figure 8A), hypertrophy/hyperplasia of Bowman’s capsule (Figure 8C) and generalized interstitial hyperplasia in the kidney (Figure 8 A–C). *Parvicapsula minibicornis* was found by ISH in both glomeruli and the lumen of renal tubules (Figure 8D).

#### Ichthyophonus hoferi

Two of five samples showed fungal cysts in the endocardium, sometimes associated with nodules of granulomatous inflammation (Supplementary Material, Fig. S2A). *I. hoferi* was clearly identified in the center of the granulomatous lesions in every organ infected, although the inflammatory reaction was not always associated with the fungal cysts (Supplementary Material, Fig. S2B).

#### Ceratonova shasta

One of six fish examined for *C. shasta* had moderate chronic enteritis (Supplementary Material, Fig. S3A), and one showed mild inflammation and proliferation of the epithelial cells of the gills from the developing stage of *C. shasta* (Supplementary Material, Fig. S4B). *Ceratonova shasta* was detected in the host lamina propria of the intestine (Supplementary Material, Fig. S3B), while some immature forms were detected in the host gill tissue (Supplementary Material, Fig. S4C and S4D).

## Discussion

Identifying physiological correlates of infection intensity in wild salmon is a means of identifying pathogens with the potential to impact early marine survival. Ours is the first study to combine transcriptional, metabolic and ionic and histopathological data to examine infectious agents carried by wild salmon. For three infectious agents recently discovered to be relevant to salmon health, including ‘*Ca.* B. cysticola’ (Gjessing *et al*., 2021), *P. pseudobranchicola* (Karlsbakk *et al*., 2002; Nylund *et al*., 2005) and PRV (Di Cicco *et al*., 2018; Polinski *et al*., 2021), this is the first study to report associations with physiological condition in wild fish. We provided the first evidence of the potential impacts of PRV on both host gene expression and tissue pathology in wild juvenile Chinook salmon, findings that were highly consistent with observations in cultured fish of the same species (Di Cicco *et al*., 2018). We also provide the first evidence that two agents known to cause disease in freshwater, *C. shasta* and *P. minibicornis*, are also associated with pathology in fish caught in the marine environment, some sampled after several months at sea.

Typically, studies investigating correlations between physiology and infectious agents are conducted under controlled conditions in laboratories. However, many of the agents we investigated have not been cultured previously, and to conduct such a controlled experiment across so many agents at once would require vast resources. The value of a study such as ours is the ability to test for physiological associations with levels of infection ecologically relevant to wild salmon across many agents at once. Results from such a study can prioritize pathogens that may warrant further investigation using controlled experiments capable of causal inference. Some negatives of our approach are that acute infections are difficult to detect since highly impacted fish are rarely encountered (Bakke and Harris, 1998), and there are many non-pathogen sources of variation that confound potential associations. Furthermore, while co-infections are likely playing an important role in physiological responses (Kotob *et al*., 2017), we are limited in our ability to explore them. Nonetheless, by looking across dozens of genes, five plasma variables and histopathology, we were able to consider the weight of evidence when determining which pathogens may warrant further study.

Of all the plasma variables and gene expression groups that we considered, none had more associations across pathogens than the gill osmoregulation genes. PC1 for gill osmoregulation was negatively associated with five pathogens and total pathogen taxa and strongly positively associated with *P. pseudobranchicola* load. A closer investigation of the genes involved (Supplementary Material, Fig. S5) revealed that genes coding components of sodium potassium adenosine triphosphate (Na,K-ATPase) in gill were those frequently negatively associated with pathogen load (although not for *P. minibicornis*). Such a relationship has been observed previously where Amoebic Gill Disease was associated with a decrease in Na,K-ATPase, likely due to the reduction in chloride cells caused by the host response to infection (Nowak *et al*., 2013; Chang *et al*., 2019). It is possible that pathogens with negative associations with expression of Na,K-ATPase isoforms in our study cause similar damage, however we conducted histopathology on only a subset of these (*C. shasta* did show some evidence of gill lesions and also a negative association with gill osmoregulation).

Plasma glucose, the only plasma variable not to show considerable deviations from literature baseline values (Supplementary Material, Table S1), shared a similar pattern of correlations with gill osmoregulation. *Parvicapsula minibicornis* and *C. shasta* infections were negatively associated with plasma glucose levels in our study. In addition, both RIB and total pathogen taxa were negatively associated with glucose. Starvation can lead to depressed plasma glucose levels (Furné *et al*., 2012), and a commonly observed clinical sign of disease for many pathogens is a reduction or cessation of feeding (Conte, 2004), sometimes accompanied by anorexia (Nowlan *et al*., 2020). For example, Mesa et al (Mesa *et al*., 2000) found that Chinook salmon experimentally infected with *Renibacterium salmoninarum* exhibited reduced plasma glucose levels, as well as lower mass, relative to controls. *Parvicapsula pseudobranchicola* was the only pathogen demonstrating a positive association with plasma glucose. In a recent study (Bass *et al*., 2022), *P. pseudobranchicola* had a strong, positive association with mass at length for Chinook salmon, suggesting that highly infected fish were above average weight for their length. One interpretation of the gill osmoregulation and plasma glucose results in combination is that, for some pathogens, highly infected fish are experiencing impacts to ionoregulatory mechanisms in the gill and as a result (or concurrently) are less capable of foraging. We note that this pattern occurred for the total pathogen taxa metric, one of our variables that was representative of coinfection. This variable has been previously associated with decreased population-level survival and elevated predation (Miller *et al*., 2014; Furey *et al*., 2021; Bass *et al*., 2022).

For almost every pathogen with significant physiological associations, correlations between pathogen load and gene expression differed dramatically between gill and liver tissue, leading to separate clusters for liver and gill gene groups (Figure 4). The notable exception to this rule was the strong upregulation of VDD genes in both gill and liver tissue associated with PRV load, but this is unsurprising given how organisms systemically respond to viruses (Miller *et al*., 2017). We suspect that this dichotomy in gene expression between tissues is a result of tissue tropism, for instance, with known gill parasites `*Ca.* B. cysticola’ and *P. pseudobranchicola* (primarily a parasite of the pseudobranch but also infecting and damaging gills (Nylund *et al*., 2018)) showing strong upregulation of gill immune gene expression. The diverse mechanisms through which pathogens interact with host physiology likely resulted in a complex array of responses in our study, limiting our ability to generalize host response across pathogens (Holzer *et al*., 2021). Furthermore, by limiting our molecular tests to gill and liver tissue, we could be missing important associations between pathogens and physiological responses, for example in the case of *Myxobolus arcticus* which targets brain tissue or *C. shasta* which targets the intestine (although histology did reveal that *C. shasta* was the only pathogen tested that showed pathology in the gastrointestinal tract).

We observed few strong associations between blood plasma variables and pathogen loads, which could be due to the fact that many of the plasma variables we measured are highly sensitive to the acute stress response which is triggered by capture and handling (Donaldson *et al*., 2014). The time from the initiation of the capture experience to blood sampling ranged from 15 to 60 min, and throughout this range, some increase in several of the plasma variables will occur (e.g. lactate). As a result, abnormal concentrations of any of these plasma variables caused by infectious agent pathology would likely be overwhelmed by large deviations from homeostasis caused by the acute stress response and thus unlikely to be observed. The exception to this pattern may be plasma glucose, the elevation of which is a secondary response to acute stress that ensues around 2 hours after a stressor is applied (Pickering *et al*., 1982; Dick *et al*., 2018). Researchers conducting similar studies in the future should weigh the utility of plasma variables given their reactivity to capture stress and carefully select parameters featuring slower response times to acute stress.

Wild fish are rarely collected in a symptomatic disease state and thus histology is not routinely used for observational studies of wild fish such as ours. Because fish in an advanced disease state are unlikely to survive in the presence of predators, wild fish usually present with only mild to moderate lesions, making the observation of tissue modifications due to pathological processes very challenging to detect. Regardless, histopathological observations of a disease agent and associated tissue damage represent the ultimate evidence of the physiological effect of infectious agents in the host. By applying ISH in this study, which allowed us to localize specific agents in tissues, we were able to confirm the association between specific agents and corresponding lesions observed in the fish tested.

### Potential physiological impacts of select infectious agents

#### Piscine orthoreovirus

Despite only nine fish with detections, PRV demonstrated the strongest associations with gene expression in the study, consistent across both gill and liver tissues. PRV was recently identified as one of the pathogens most likely to be associated with decreases in survival and condition for Chinook and Coho salmon in British Columbia (Bass *et al*., 2022). Our study demonstrates that PRV occurs in wild Chinook salmon in their first marine year in British Columbia, and that these fish have gene expression patterns indicating a response to viral infection in multiple tissues, as well as cellular changes consistent with PRV-related jaundice/anemia.

The relationship between PRV and the activation of the VDD genes was first observed in samples from a Chinook salmon farm outbreak of jaundice/anemia (aka jaundice syndrome) (Miller *et al*., 2017) and confirmed in a subsequent independent assessment of audit samples (Di Cicco *et al*., 2018). Importantly, Di Cicco *et al*. (2018) showed that the VDD response did not appear to activate in the host until the virus was observed in the extracellular space outside of its primary infective tissue, red blood cells (RBCs), likely due to a massive hemolysis. Unlike in Atlantic salmon, Chinook salmon infected with PRV could present anemia (Miller *et al*., 2017; Di Cicco *et al*., 2018) and show a threshold response between PRV load and VDD activation potentially suggesting a greater vulnerability in Chinook than Atlantic salmon to the PRV-1a variant in BC (Di Cicco *et al*., 2018). The innate antiviral response induced by salmonids in the presence of PRV was recently described by Dahle *et al*. (2022), who listed immune components that were upregulated in the presence of PRV infection, including CD4, CD8, IgMs, MHCI, MHCII, IRF, STAT1, IFNa and Mx, many of which we included as assays in this study (Supplementary Material, Fig. S5 and S6). For the majority of these genes we observed significant positive associations with PRV load, particularly in liver tissue (Supplementary Material, Fig. S6). Dahle *et al*. (2022) suggest that a strong innate immune response may be associated with pathological outcomes although there is currently no evidence that these immune mechanisms offer protection against PRV-associated pathological changes.

In our study, loads of PRV were overall lower compared with the farm audit Chinook salmon presented in Di Cicco *et al*. (2018). This finding was expected as the wild fish were sampled live, indicating an overall early phase of the infection, while the farm audit fish, in which high PRV load is usually observed, were all moribund or dead fish. However, in the present case, the VDD signal was still strongly associated with PRV load in liver and gill. Di Cicco *et al*. (2018) showed that milder lesions associated with earlier stages of the development of jaundice/anemia disease are present in fish that did not yet contain the clinical signs of jaundice (external yellowing) or anemia (pale gills), but only in fish classified as VDD+. Similar lesions occurring in the presence of PRV, affecting primarily the heart (mild endo/myocarditis), the spleen (congestions and accumulation of hemosiderin) and the kidney (hemopoietic tissue hyperplasia and renal tubule necrosis), were observed in the juvenile chinook included in the present study. We therefore suspect that these fish were in an early stage of development of jaundice/anemia. Whether wild Chinook salmon with a late-stage disease would survive long enough to be sampled, and if they are physiologically compromised at early stages of disease development, are certainly questions worth pursuing in the future.

Importantly, all of the Chinook with high loads of PRV showing signs of disease in this study were sampled in the cool fall/winter period, the same temporal time that jaundice/anemia occurs in Chinook salmon on farms (Di Cicco *et al*., 2018) and Heart and Skeletal Muscle Inflammation occurs in Atlantic salmon (Di Cicco *et al*., 2017). While two PRV challenge studies have been carried out in Chinook salmon from the Pacific Northwest, both studies were not carried out in temperatures typically experienced overwinter and focused on recapitulation of clinical signs of disease (mortality, jaundice) rather than recapitulation of the pathological lesions that lead to clinical manifestations (Garver *et al*., 2016; Purcell *et al*., 2020). Given that most PRV challenge studies worldwide have successfully demonstrated the pathology but failed to induce disease powerful enough to induce clinical signs, including mortality, the failure of these studies to demonstrate a cause-and-effect relationship with “disease” was unsurprising. Future studies need to feature low-temperature trials and focus on pathological outcomes, as described in Di Cicco *et al*. (2018).

#### *‘Candidatus* Branchiomonas cysticola’

The bacterium *‘Candidatus* Branchiomonas cysticola’ is commonly found in gill epitheliocysts in farmed Atlantic salmon (Toenshoff *et al*., 2012; Mitchell *et al*., 2013) and was recently identified as a major contributor to inflammatory gill disease in that species (Gjessing *et al*., 2021). However, some have suggested that, as this bacterium is a member of the fish gill microbiota in healthy fish, it may not be pathogenic (Gunnarsson *et al*., 2017). Mitchell *et al*. (2013) posited that, although *‘Ca.* B. cysticola’ loads in Atlantic salmon were positively associated with the severity of gill inflammation, the negative impacts may be load-dependent or more likely to cause disease when additional agents are present—thus providing two explanations for why many infections of this high-prevalence agent may not be impactful to the host.

Consistent with the theory that this bacterium may impact gills, we observed several positive associations between immune gene groups and ‘*Ca.* B. cysticola’ load in gill tissue but no associations in liver tissue. Specifically, *‘Ca.* B. cysticola’ load was positively associated with the expression of innate and adaptive immunity as well as immune cell signaling, with a strong activation of inflammatory genes including CD8, MMP25, MMP13, IL-1b, IL-8 and SAA (Supplementary Material, Fig. S5). We also observed a positive association between ‘*Ca.* B. cysticola’ load and plasma lactate, potentially a result of earlier onset of anaerobic metabolism during capture due to decreased gas exchange by inflamed gill tissue. Finally, *in situ* hybridization localized ‘*Ca.* B. cysticola’ to epitheliocysts in two Chinook salmon. These results indicate that, similarly to Atlantic salmon, *‘Ca.* B. cysticola’ is found in gill epitheliocysts of Chinook salmon and likely triggers a pro-inflammatory immune response in the gill tissue of this Pacific species which may result in impacted gill function.

Although in previous research ‘*Ca.* B. cysticola’ has been highly prevalent in out-migrating salmonid smolts (Healy *et al*., 2018; Stevenson *et al*., 2020) and adult Chinook salmon returning to spawn (Bass *et al*., 2017, 2019), no studies of this agent in wild fish have revealed associations with survival. By acquiring fish after they left the freshwater environment but before they matured, our study focused on a different life stage from these previous studies. Studying the same life stage, Tucker *et al*. (2018) found that declines in prevalence were coupled with load truncation, a combination of characteristics consistent with pathogen-mediated mortality. Bass *et al*. (2022) found that in the fall and winter, Chinook body mass was negatively associated with ‘*Ca.* B. cysticola’ load, indicating that as infection intensity increased, fish became more underweight (although the opposite was true in the spring and summer). The findings from the two aforementioned studies coupled with the physiological findings from our current study suggest that although ‘*Ca.* B. cysticola’ appears extremely common in Pacific salmon, further research is needed to determine whether under stressful environmental conditions, this bacterium may act cumulatively or synergistically to reductions in the health and survival of wild salmon.

#### Parvicapsula pseudobranchicola

Correlations between the load of this highly prevalent marine myxozoan and gene expression (and to a lesser extent, blood parameters) were very similar to those we described above for the bacterium ‘*Ca.* B. cysticola’. In addition to associations in gill tissue genes suggestive of immune response and inflammation, there was a significant positive association between *P. pseudobranchicola* load and osmoregulatory gene expression (unique in the study). Plasma glucose, lactate and sodium also increased with *P. pseudobranchicola* load but not significantly after FDR correction.

Much of what is known regarding the biology of this pathogen comes from a single longitudinal study of Atlantic salmon held in aquaculture pens in Norway (Nylund *et al*., 2018). In that study, *P. pseudobranchicola* was 100% prevalent in gills 49 days after introduction of juvenile salmon to seawater and PCR-measured loads in gill were highly correlated with loads in pseudobranchs. Because previous work had not led us to anticipate the many correlations between *P. pseudobranchicola* load and gene expression, we did not conduct histopathology targeting this agent. However, the presence of many significant correlations between *P. pseudobranchicola* load and gene expression suggest that gill tissue, in comparison to liver, is the more likely location where related impacts occur. While Nylund *et al*. (2018) found that the primary tissue infected by *P. pseudobranchicola* in Atlantic salmon was the pseudobranch, the only other tissue where the parasite was localized using ISH was the gill. We did not sample pseudobranchs for any purpose in this study, but these organs are adjacent to the gills.

Nylund *et al*. (2018) observed an increase in *P. pseudobranchicola* spores and normalized expression (via PCR) in Atlantic salmon pseudobranchs until around day 147 following seawater transfer, after which both of these measures decreased dramatically, likely due to the parasite rupturing from pseudobranch cells and dispersing into the surrounding environment. Such concentrated development and subsequent tissue damage is likely to trigger the up-regulation of immune-related genes. Thus, if the trajectory of the pathology of the parasite is similar in Chinook salmon, the positive association between gill genes associated with immune response and inflammation that we observed might be expected. Using the same PCR platform used in our study, Deeg *et al*. (2022) observed positive associations between *P. pseudobranchicola* load and the expression of immune- and inflammation-related genes in the gill tissue of pink and sockeye salmon collected in the Gulf of Alaska. Given that *P. pseudobranchicola* was one of the agents with the most associations in our study, that it appeared important for Deeg *et al*. (2022), and that it had high consistency in associations with survival between Chinook and Coho salmon in a recent epidemiological study (Bass *et al*., 2022), we suggest that this marine parasite should receive further detailed study in Pacific salmon.

#### Parvicapsula minibicornis

*Parvicapsula minibicornis* is a myxozoan parasite that targets the glomeruli of the kidney, thus compromising host osmoequilibrium and sometimes resulting in mortality for adult salmon (Bradford *et al*., 2010b). Accordingly, in adult sockeye salmon conducting freshwater migrations, plasma osmolality was negatively correlated with *P. minibicornis* abundance in kidney tubules (Bradford *et al*., 2010a). In our study, evidence of damage caused by *P. minibicornis* was observed in histopathology, including lesions in the kidney characteristic of *P. minibicornis* pathology and localization of the pathogen in these tissues via ISH. In gill tissue, another component of the osmoregulatory system, genes associated with osmoregulation were down-regulated. Perhaps as an outcome of these impacts on multiple aspects of the osmoregulatory system, we found a positive association (prior to FDR correction) between plasma osmolality and *P. minibicornis* load. This is the opposite result found by Bradford *et al*. (2010a), likely because in this case fish were collected in saltwater. To date, all studies linking *P. minibicornis* to disease have focussed on returning adult salmon in freshwater (e.g. Bradford *et al*., 2010). However, the co-localization of lesions in the host kidney with *P. minibicornis* and the stress-related signals we discovered in the host genes indicate the high likelihood that this parasite is capable of causing disease in juvenile salmon in the ocean.

Myxozoans are a broad group of multicellular parasites with a diversity of life history strategies and methods for evading host immune systems (Holzer *et al*., 2021). We observed a widespread downregulation of host gill genes involved with intracellular immunity and inflammation associated with increasing loads of *P. minibicornis*. Similar to our study, experimental myxozoan infections in gilthead sea bream and rainbow trout have resulted in broad downregulation of immune-related genes (Davey *et al*., 2011; Barrett and Bartholomew, 2021). In contrast, *P. pseudobranchicola* appeared to show patterns of gene expression complementary to those of *P. minibicornis* in nearly all cases. This opposite pattern of molecular response could be driven by pathogen tissue tropism. Of 14 fish receiving histopathological sampling targeting *P. minibicornis* in this study, one fish showed pathology in the gills while five showed pathology in kidney (Supplementary Material, Table S3). These results are consistent with those of Bradford *et al*. (2010b), who showed that adult sockeye in freshwater experienced higher *P. minibicronis* loads in kidney before gills. Thus, immune function in gills could be less of a priority for a resource limited host battling *P. minibicornis* infection in other tissues relative to a host contending with *P. pseudobranchicola* in gills and the adjacent pseudobranchs. Alternatively, it is possible that although these two myxozoans are classified in the same genus, they may have completely different approaches to evading the immune response of the host organism (Holzer *et al*., 2021).

#### Loma salmonae

This marine microsporidian was the only pathogen that demonstrated a stronger gene expression response in liver tissue compared to gill tissue. This result is unexpected as gill is the target tissue for the formation of cyst-like xenomas that eventually rupture and induce chronic inflammation and stress in the host. Additionally, liver tissue has not been demonstrated as a target tissue through molecular methods or histology (Speare *et al*., 2012). Nevertheless, we saw positive associations between *L. salmonae* load and several MRS genes in liver tissue. Components of adaptive immunity and immune cell signaling were also positively associated with *L. salmonae* load. Although long considered an organ for metabolism and energy storage, teleost livers do show some immune response to pathogens (Causey *et al*., 2018).

#### Ichthyophonus hoferi

*I. hoferi* is a mesomycetozoan parasite of over 100 species of fish across marine, brackish and freshwater habitats (McVicar, 2011). It was prevalent among returning Chinook salmon in the Yukon River and was suspected of causing pre-spawn mortality (Kocan *et al*., 2004). In our study, we observed cysts typical of the parasite, localized primarily in the heart, associated with a mild to moderate granulomatous inflammatory reaction in areas where the cysts were damaged. We observed an immune stimulation of the hemopoietic tissue of the kidney, but *I. hoferi* cysts and other immature stages of the parasite were not visible in the histology sections, and only very rare granulomata were observed through ISH in the kidney, with no other microscopic cysts or “free”, immature stages of the agent present in the tissue. A weak but consistently positive upregulation across multiple gene groups in gill tissue was observed.

#### Ceratonova shasta

*Ceratonova shasta* is a myxozoan parasite of fish intestines, and it is commonly found in Chinook salmon in several large river systems from BC to California (Fujiwara *et al*., 2011). The similar life history shared by *P. minibicornis* and *C. shasta* (Bartholomew *et al*., 2006) and the fact that 82% of *C. shasta* positive fish were also positive for *P. minibicornis* probably partially explained the similar patterns between these two agents and gene expression (Figure 4). We found minimal associations between *C. shasta* and physiological disturbance at the molecular or protein level, but histology revealed moderate inflammatory lesions in the gastrointestinal system and in the tips of gill lamellae where developing *C. shasta* spores were forming*.* Osmoregulatory genes in gill appeared slightly down-regulated, perhaps due to gill lesions caused by the presence of *C. shasta.* Although *C. shasta* is widely considered a threat to Chinook salmon in freshwater environments, these results indicate that fish carrying *C. shasta* infections as they leave freshwater may experience physiological impacts in the ocean—a potential example of a pathogen-mediated carryover effect. Recent studies have indicated that *C. shasta* infections are associated with reduced weight of Chinook salmon during freshwater migrations (Mauduit *et al*., 2022) and soon after ocean entry (Bass *et al*., 2022).

### Conclusions

Through the combination of transcriptional, metabolic and ionic and histopathological data, we identified several infectious agents with multiple lines of evidence suggesting physiological impact to wild Chinook salmon with increasing pathogen loads. Our results support the use of molecular methods to monitor the impact of infectious agents on wild populations, which can be applied alongside regular molecular monitoring of infectious agents among Pacific salmon. Once a more definitive relationship between infection, disease and survival is established, incorporation of infectious agents and host transcriptome may enhance the accuracy of models estimating survival.

We found that wild Chinook salmon with PRV demonstrated a molecular response to viral disease development and pathology consistent with jaundice/anemia in farmed Chinook salmon (Di Cicco *et al*., 2018) as well as similar diseases in Pacific salmon caused by other strains of PRV (reviewed in Di Cicco *et al*., 2018). These findings, coupled with the higher PRV infection rates for populations exposed to salmon farms (Mordecai *et al*. 2021) and evidence indicating population-level impacts (Bass *et al*., 2022), highlight the threat PRV may pose to wild Chinook salmon populations and the need for precaution in our interpretation of risks related to PRV. To conclusively determine whether PRV (or other pathogens of concern) impacts wild or hatchery salmon populations, well-designed paired-release studies featuring experimental manipulations that alter host resistance to infection (i.e. vaccines, which have not yet been developed to scale (Dahle *et al*., 2022)) are necessary (e.g. Vollset *et al*. (2018)).

Southern British Columbia Chinook salmon populations have declined to record low levels (COSEWIC, 2019). Our group’s research has sought to build a better understanding of the role that infectious disease may play in these declines. Although this study did not account for the many other factors impacting the survival of salmon populations, we acknowledge that some of these may interact with infectious disease in important ways. For instance, rising temperatures alter the transmission, development and pathogenicity associated with many infectious agents and can change host susceptibility (Harvell *et al*., 2002; Lafferty, 2009; Miller *et al*., 2014). Also, predation in the marine environment is an important source of mortality and poor physical condition resulting from immune suppression and susceptibility to infections might make salmon more likely to become prey (Miller *et al*., 2014; Furey *et al*., 2021). Future studies need to continue to explore the cumulative or synergistic relationships among salmon conditions, infections, diseases and environmental changes in order to better understand the causes of the decline of this imperilled group of species.

## Funding

This research was conducted as one component of the Strategic Salmon Health Initiative (SSHI), co-funded by Genome British Columbia; Pacific Salmon Foundation; Fisheries and Oceans Canada; and the Natural Sciences and Engineering Research Council of Canada. Funding was provided to YW through a Mitacs Accelerate Internship.

## Conflicts of Interest

The authors have no conflicts to declare.

## Data availability

Data used in analyses and R code for statistical analyses are provided as supplementary material.

## Author Contributions

The study was conceived and designed by KM and SH. Field collections were overseen by DP. Molecular analyses were performed by KK, TM and SL. Histological analyses were conducted by EC and HF. Blood chemistry analyses were conducted by YW and DP. Statistical analyses were designed and conducted by YW and AB. Writing was led by YW and AB with contributions from all authors.

## Ethics Statement

All samples, including fish tissue and blood, were collected under a scientific fishing permit (MECTS #2014-502-00249) issued to Pacific Region Department of Fisheries and Oceans (DFO) staff by the Government of Canada, DFO, Regional Director Fisheries Management. This work does not require an animal care protocol pursuant to an exemption contained in the Canadian Council on Animal Care (CCAC) guidelines applying to fish lethally sampled under government mandate for assessment purposes (4.1.2.2).

## Supplementary Material

Web_Material_coad031
