# Supplementary material for: Infectious agents and their physiological correlates in early marine Chinook salmon (Oncorhynchus tshawytscha)
Source: Conserv Physiol. 2023 May 19;11(1):coad031. doi: 10.1093/conphys/coad031 (PMC10494280; doi:10.1093/conphys/coad031)
Supplement: Web_Material_coad031 [file web_material_coad031.zip › supplement to YW microbes Cons Phys revised.pdf]

Supplementary Material

Table S1: Mean (± SD) plasma variables for Chinook salmon collected around Vancouver Island (WCVI = West Coast Vancouver Island, SEVI = Southeast Vancouver Island, NEVI = Northeast Vancouver Island; see Figure 1) by trawl capture (lower rows of table) along with "baseline" values from select publications (upper rows). Blood was drawn within 30 min of landing but trawls could be 15-30 min in duration. Sample size (N) is given for subyearling (SY) and yearling (Y) Chinook salmon.

**Source**	**Season**	**Capture region**	**N (SY/Y)**	**Fork length (mm ± SD)**	**Mass (g ± SD)**	**Lactate (mmol/L)**	**Glucose (mmol/L)**	**Chloride (mmol/L)**	**Sodium (mmol/L)**	**Osmolality (mOsm/kg)**
(Casanovas *et al.*, 2021)			**223-256/0**	**70 - 689**	**44 - 3164**			**128.2 - 140.6**	**151.8 - 163.9**	
(Wagner *et al.*, 1969)										**348 ± 20**
(Barton *et al.*, 1986)				**206**	**97.9**	**2.2**	**4.3**		**154.2**	
(Blackburn, 1987)*			**60/0**						**162**	**323-372***
(Braceland *et al.*, 2017)[&]					**3500**		**4 - 6.2**[&]	**128 - 136**[&]	**158 - 170**[&]	
(Parker and Black, 1959)[+]			**16**		**3600-9100**	**3.6 ± 2.1**<br>**8.3 ± 2.5**				
**This study**	**Summer**	**WCVI**	**19 / 8**	**201.4 ± 48.1**	**118.3 ± 72.4**	**15.4 ± 2.5**	**2.5 ± 1.0**	**170.5 ± 15.1**	**182.6 ± 13.8**	**406.5 ± 30.2**
		**SEVI**	**33 / 13**	**156.2 ± 29.9**	**49.2 ± 37.2**	**12.9 ± 2.4**	**3.2 ± 1.0**	**144.7 ± 15.8**	**160.0 ± 15.3**	**359.1 ± 29.1**
		**NEVI**	**4 / 6**	**186.8 ± 56.8**	**99.3 ± 56.8**	**13.2 ± 0.9**	**2.7 ± 0.9**	**167.1 ± 16.2**	**179.3 ± 10.9**	**382.1 ± 19.1**
	**Fall**	**WCVI**	**19 / 5**	**208.7 ± 40.1**	**122.9 ± 80.7**	**17.1 ± 3.0**	**3.7 ± 1.8**	**160.8 ± 14.4**	**183.8 ± 17.0**	**389.4 ± 28.2**
		**SEVI**	**3 / 2**	**183.2 ± 36.4**	**76.2 ± 49.8**	**13.2 ± 2.9**	**3.9 ± 1.2**	**171.8 ± 17.6**	**171.1 ± 20.6**	**384.6 ± 34.9**
		**NEVI**	**18 / 24**	**225.5 ± 56.1**	**160.9 ± 155.9**	**14.2 ± 2.0**	**3.2 ± 1.0**	**155.1 ± 14.4**	**173.5 ± 10.8**	**380.7 ± 30.5**
	**Winter**	**WCVI**	**43 / 4**	**217.7 ± 26.8**	**120.9 ± 46.6**	**10.1 ± 2.5**	**3.9 ± 0.8**	**150.9 ± 16.0**	**166.6 ± 11.3**	**355.9 ± 30.4**

		**SEVI**	**9 / 0**	**276.1 ± 24.0**	**260.4 ± 66.7**	**16.4 ± 1.8**	**3.5 ± 1.4**	**142.2 ± 5.0**	**170.6 ± 5.4**	**364.1 ± 12.9**
		**NEVI**	**1 / 0**	**268.0 ± NA**	**235.0 ± NA**	**10.9 ± NA**	**3.0 ± NA**	**133.1 ± NA**	**152.2 ± NA**	**325.0 ± NA**

***** = experiment conducted with Coho salmon

[&] = experiment conducted with Atlantic salmon

[+]= top value from fish sampled 0-0.5 hr after troll capture, bottom value from those sampled 1-1.5 hr after troll capture

References for Table S1

Barton BA, Schreck CB, Sigismondi LA (1986) Multiple acute disturbances evoke cumulative physiological stress responses in juvenile chinook salmon. *Transactions of the American Fisheries Society* 115: 245–251.

Blackburn J (1987) Revised procedure for the 24-hour seawater challenge test to measure seawater adaptability of juvenile. *Can Tech Rep Fish Aquat* 1515.

Braceland M, Houston K, Ashby A, Matthews C, Haining H, Rodger H, Eckersall PD (2017) Technical pre-analytical effects on the clinical biochemistry of Atlantic salmon (Salmo salar L.). *Journal of Fish Diseases* 40: 29–40.

Casanovas P, Walker SP, Johnston H, Johnston C, Symonds JE (2021) Comparative assessment of blood biochemistry and haematology normal ranges between Chinook salmon (Oncorhynchus tshawytscha) from seawater and freshwater farms. *Aquaculture* 537: 736464.

Parker RR, Black EC (1959) Muscular fatigue and mortality in troll-caught chinook salmon (Oncorhynchus tshawytscha). *Journal of the Fisheries Board of Canada* 16: 95–106.

Wagner HH, Conte FP, Fessler JL (1969) Development of osmotic and ionic regulation in two races of chinook salmon Oncorhynchus tshawytscha. *Comparative Biochemistry and Physiology* 29: 325–341.

Table S2: Primer and probe sequences corresponding to assay for infectious agents and biomarkers used in HT-qPCR analyses on juvenile Chinook salmon *(Oncorhynchus tshawytscha).* Amplification factor with * sign indicates this assay was excluded from statistical analysis for both gill and liver tissue due to an unacceptable amplification factor. Amplification factor with ** sign indicates this assay was excluded from statistical analysis only for liver tissue due to a large number of missing values in the samples

**Symbol**	**Infectious agent/<br>Host gene name**	**Assay<br>Class**	**Forward Primer Sequence (5'-3'),<br>Reverse Primer Sequence (5'-3'),<br>Probe Sequence (FAM-5'-3'-MGB)**	**Accession number**	**Amplification Factor**
52Ro	52 kDa Ro protein-2	Host gene	F: TGCACTATTGCCCAGTAACCAT<br>R: TGCAAGAGGAGATGCCAACA<br>P: AGTAGGATTCACAGAGAGTT	CX141267	1.95
ACTB	Beta actin	Host gene	F: GAAATCGCCGCACTGGTT<br>R: CGGCGAATCCGGCTTT<br>P: TTGACAACGGATCCGGT	BG933897	2.15
ALDOA	Aldolase A	Host gene	F: CGTGATTCAGTGTTGTCATCTTGA<br>R: TTCCTCCAGTGTTTTTTTCAGTCA<br>P: AAGTACATGTGCCTTCTT	C205R054,<br>C063R133	2.19
B2M	Beta-2-Microglobulin	Host gene	F: TTTACAGCGCGGTGGAGTC<br>R: TGCCAGGGTTACGGCTGTAC<br>P: AAAGAATCTCCCCCCAAGGTGCAGG	AF180490	2.88*
C1Qc	Complement C1q C Chain	Host gene	F: CGCCGGTGAGTGGAATCTA<br>R: CTTCTCCATCATGTGGTGTGCTA<br>P: ACCTCCAAACATAGAAGAG	AM042158	1.96
C3	Complement factor 3	Host gene	F: ATTGGCCTGTCCAAAACACA<br>R: AGCTTCAGATCAAGGAAGAAGTTC<br>P: TGGAATCTGTGTGTCTGAACCCC	U61753,<br>AF271080	2.04
C7	Complement factor 7	Host gene	F: ACCTCTGTCCAGCTCTGTGTC<br>R: GATGCTGACCACATCAAACTGC<br>P: AACTACCAGACAGTGCTG	CA052045	2.43*
CA054694	Mitochondrial ribosomal protein	Host gene	F: CCACCTGAGGTACTGAAGATAAGACA	CA054694	1.96

Symbol	Infectious agent/<br>Host gene name	Assay Class	Forward Primer Sequence (5'-3'),<br>Reverse Primer Sequence (5'-3'),<br>Probe Sequence (FAM-5'-3'-MGB)	Accession number	Amplification Factor
	(VAR1)		R: TTAAGTCCTCCTTCCTCATCTGGTA		
			P: TCTACCAGGCCTTAAAG		
CA4	Carbonic anhydrase 4	Host gene	F: GGTCATTTTGGTTTTGTACACAGTCT	C148R144	1.98
			R: CCTAGATATAGCTATCCACGTACTCACCTA		
			P: TGATACGTGGTATAGAAAAG		
CCL4	Chemokine (C-C motif) ligand 4	Host gene	F: TCTCTTCATTGCAACAATCTGCTT	C240R068	1.98
			R: ACAGCAGTCCACGGGTACCT		
			P: CTACGCAGCAGCATT		
CD4	cluster of differentiation 4	Host gene	F: CATTAGCCTGGGTGGTCAAT	AY973028	1.90
			R: CCCTTTCTTTGACAGGGAGA		
			P: CAGAAGAGAGAGCTGGATGTCTCCG		
CD83	cluster of differentiation 83	Host gene	F: GTGGCGGCATTGCTGATATT	CF752979	2.04
			R: CTTGTGGATACTTCTTACTCCTTTGCA		
			P: CACCATCAGCTATGTCATCC		
CD8a	cluster of differentiation 8 subunit α	Host gene	F: ACACCAATGACCACAACCATAGAG	AF178054	1.99
			R: GGGTCCACCTTTCCCACTTT		
			P: ACCAGCTCTACAACTGCCAAGTCGTGC	CA064247	
CD9	cluster of differentiation 9	Host gene	F: CTTGATCTGTTTCATGAGGATGCT		2.00
			R: ACCTCCTCCTGTTGCTCCTAGA		
			P: CAGCACACCAGGGC		
CFTR-I	Cystic fibrosis transmembrane conductance regulator I	Host gene	F: GAGCTGTCAGAGAGGAAGTTCTCA	C161R157	1.95
			R: GCAGCGACTCTTCAACCTGAT		
			P: TGGTGCCCGAGGAC		
CIRBP	Cold-inducible RNA-binding protein	Host gene	F: GGGATGGTGGAGACCTTCTCT	C026R122,	N/A*
			R: CAGAACCCACAGCGATCCTAA	C251R142,	
			P: TTCTCTAGTCCACTGGGCT	CA048095,<br>CB499204	
COMMD7	COMM domain-containing protein 7	Host gene	F: CAAAGCCAGTATGGACTGTTTCAG	CB512167	1.98
			R: TTGTTTTCTGCTGCCCCTCTA		
			P: ACCTGATCGCCAGTAGCATGAGCATGTAC		
COX6B1	Cytochrome c oxidase subunit 6B1	Host gene	F: GCCCCGTGTGACTGGTATAAG	C193R155,	2.00
			R: TCGTCCCATTTCTGGATCCA	C074R104,	

Symbol	Infectious agent/ Host gene name	Assay Class	Forward Primer Sequence (5'-3'), Reverse Primer Sequence (5'-3'), Probe Sequence (FAM-5'-3'-MGB)	Accession number	Amplification Factor
			P: TCTACAAATCACTGTGCCC	C042R086, C149R170	
DEXH	ATP-dependent RNA helicase	Host gene	F: CCATAAGGAGGGTGTCTACAATAAGAT	FN396359	1.94
			R: CTCTCCCCCTTCAGCTTCTGT		
			P: TGGCGCGCTACGTG		
EF-2	Elongation factor 2	Host gene	F: AGGTCACAGCCGCCCTTAG	C262R107,	1.99
			R: ACACAGTCTCTGTCTGCACACACA	CB498321	
			P: CGACTGCGTCTCAGGT		
FK506	FK506-binding protein 10 precursor	Host gene	F: ACTATGAGAATGCCCCCATCAC	C067R011	N/A*
			R: CTCGTCCAGACCCTCAATCAC		
			P: CCTGGGAGCCAACAA		
FYB	FYN-T-binding protein	Host gene	F: TGCAGATGAGCTTGTTGTCTACAG	CA053392	1.92
			R: GCAGTAAAGATCTGCCGTTGAGA		
			P: CTCAACGATGACATCCACAGTCTCCCC		
GAL3	Galectin-3-binding protein precursor	Host gene	F: TTGTAGCGCCTGTTGTAATCATATC	CB515011	1.99
			R: TACACTGCTGAGGCCATGGA		
			P: CTTGGCGTGGTGGC		
glut2	Glucose transporter 2	Host gene	F: GGAACCTTACATCAACTGGCTACA	CB514105,	2.01
			R: GCAGTGGCCAGTAGTAGTCATTACC	CB514228,	
			P: CTGGTATACTACTGAGTCAGG	CA037334	
HBA	Hemoglobin subunit α	Host gene	F: GCCCTGGCTGACAAATACAGA		2.04
			R: GAGCAGGAACTGGAGTCCAATG	C228R104	
			P: ACCATCATGAAAGTCC		
hep	Hepcidin	Host gene	F: GAGGAGGTTGGAAGCATTGA	AF281654.1	2.01
			R: TGACGCTTGAACCTGAAATG		
			P: AGTCCAGTTGGGGAACATCAACAG		
HERC6	Probable E3 ubiquitin-protein ligase HERC6	Host gene	F: AGGGACAACTTGGTAGACAGAAGAA	CA060884	1.98
			R: TGACGCACACACAGCTACAGAGT		
			P: CAGTGGTCTCTGTGGCT		
HIF1A_3	Hypoxia-inducible factor 1-alpha	Host gene	F: CACTACAACTTCTCCTCACTCACTCTGT	C185R118,	2.06

Symbol	Infectious agent/ Host gene name	Assay Class	Forward Primer Sequence (5'-3'), Reverse Primer Sequence (5'-3'), Probe Sequence (FAM-5'-3'-MGB)	Accession number	Amplification Factor
			R: AGCAGCCAAACTATAAGATCACTGATAC	C026R129,	
			P: CTGCCCCTTTATTTGTCTC	C089R035	
HIF1A_6	hypoxia-inducible factor 1-alpha	Host gene	F: AGAGGAGGCAGTGCTGTATTCAA	CA062104	2.00
			R: GGGACAAGGCCCTCCAAT		
			P: AGGGCCCTGACCATG		
HIF1A_7	hypoxia inducible factor 1-alpha	Host gene	F: TGGCAAATCTGCCTACGAATT	C172R081,	2.11
			R: GCAGGCTCTTGGTCACATGA	C120R146	
			P: ATCATGCCCTGGACTC		
HSC70	Heat shock cognate 70 protein	Host gene	F: GGGTCACACAGAAGCCAAAAG	CA052185	2.02
			R: GCGCTCTATAGCGTTGATTGGT		
			P: AGACCAAGCCTAAACTA		
hsp90a	Heat shock protein 90 alpha	Host gene	F: ATGACCCTCAGACACACTCCAA	C182R027,	1.95
			R: CCTCATCAATACCCAGTCCTAGCT	C015R121,	
			P: CGCATCTACAGAATGA	CA062155	
HSP90ab1	Heat shock protein 90 alpha class b	Host gene	F: GACACGGTGTTGGGTTGGTT	C101R112,	1.94
			R: TTGCAGTCAACTCTCCATGCA	CB493619,	
			P: TCATGTGCAACATAACAT	CB501628, CA767842, CB502806	
HSP90alike	Heat shock protein 90 alpha	Host gene	F: TTGGATGACCCTCAGACACACT		2.10
			R: CGTCAATACCCAGGCCTAGCT	C020R155	
			P: CCGAATCTACCGGATGAT		
HTA	HIV-1 Tat interactive protein	Host gene	F: CTTGTAACAGTTCGACATGGCTTATT	CA062248	1.96
			R: TGGTGAAGCATTTCTGTATGTCAA		
			P: TCTGTACTGAGCATCCCCGCACATTACA		
IDH3B	Isocitrate Dehydrogenase 3 Beta	Host gene	F: AGAAATCTCTACCACAGCACTGTATCA	C125R081	1.96
			R: GGCACGACTCAGGACTGTGA		
			P: TGGATATCTGGCCTGTCAT		
IFI44a	Interferon-induced protein 44 alpha	Host gene	F: CGGAGTCCAGAGCAGCCTACT	GS365948	1.99
			R: TCCAGTGGTCTCCCCATCTC		
			P: CGCTGGTCCTGTGTGA		

Symbol	Infectious agent/ Host gene name	Assay Class	Forward Primer Sequence (5'-3'), Reverse Primer Sequence (5'-3'), Probe Sequence (FAM-5'-3'-MGB)	Accession number	Amplification Factor
IFIT5	Interferon-induced protein with tetratricopeptide repeats 5	Host gene	F: CCGTCAATGAGTCCCTACACATT<br>R: CACAGGCCAATTTGGTGATG<br>P: CTGTCTCCAAACTCCCA	CA051350	2.02
IFNa	Interferon alpha	Host gene	F: CGTCATCTGCAAAGATTGGA<br>R: GGGCGTAGCTTCTGAAATGA<br>P: TGCAGCACAGATGTACTGATCATCCA	AY216595	2.03
IGFBP1	Insulin-like growth factor binding protein-1	Host gene	F: GGGTCCCTGCCACATTGAG<br>R: TTCCTGCTGAGAGCTGGTTATCT<br>P: CATGCAGCTCTGGAC	C161R148	N/A*
IgMs	Immunoglobulin	Host gene	F: CTTGGCTTGTTGACGATGAG<br>R: GGCTAGTGGTGTTGAATTGG<br>P: TGGAGAGAACGAGCAGTTCAGCA	AB044939	2.05
IgT	Immunoglobulin tau	Host gene	F: CAACACTGACTGGAACAACAAGGT<br>R: CGTCAGCGGTTCTGTTTTGGA<br>P: AGTACAGCTGTGTGGTGCA	GQ907004	2.02
IL-11	Interleukin 11	Host gene	F: GCAATCTCTTGCCTCCACTC<br>R: TTGTCACGTGCTCCAGTTTC<br>P: TCGCGGAGTGTGAAAGGCAGA	AJ535687	1.91
IL-15	Interleukin 15	Host gene	F: TTGGATTTTGCCCTAACTGC<br>R: CTGCGCTCCAATAAACGAAT<br>P: CGAACAACGCTGATGACAGGTTTTT	AJ555868.1	2.00
IL-17D	Interleukin 17D	Host gene	F: CAACAGAAGTGCGAACGATG<br>R: GATGCCACATCGCATAACAG<br>P: TGGTCGAGTATCTTTCGTGTGTTTGC	NM_001124399	2.17
IL-1B	Interleukin 1b	Host gene	F: AGGACAAGGACCTGCTCAACT<br>R: CCGACTCCAACTCCAACACTA<br>P: TTGCTGGAGAGTGCTGTGGAAGAA	AY617117	1.99
IL-8	Interleukin 8	Host gene	F: GAGCGGTCAGGAGATTTGTC<br>R: TTGGCCAGCATCTTCTCAAT<br>P: ATGTCAGCGCTCCGTGGGT	AJ310565	1.89
IRF1	Interferon regulatory factor 1	Host gene	F: CAAACCGCAAGAGTTCCTCATT	CB511515	1.85

Symbol	Infectious agent/ Host gene name	Assay Class	Forward Primer Sequence (5'-3'), Reverse Primer Sequence (5'-3'), Probe Sequence (FAM-5'-3'-MGB)	Accession number	Amplification Factor
			R: AGTTTGGTTGTGTTTTTGCATGTAG<br>P: CTGGCGCAGCAGATA		
JUN	Transcription factor	Host gene	F: TTGTTGCTGGTGAGAAAACTCAGT<br>R: CCTGTTGCCCTATGAATTGTCTAGT<br>P: AGACTTGGGCTATTTAC	CA056351	2.15
KRT8	Cyclokeratin-8	Host gene	F: CGATTGAGCGGCTGGATAA<br>R: GCATTGTTTACCTTTGACTTGAATTG<br>P: CCCCCTTCTCTACTCTCTTGCTCACCATTC	CA770356, CB492778	1.98
LdhaL	L-lactate dehydrogenase A chain-like	Host gene	F: TTTGTTTAGTGTGTGCGAGAGTTG<br>R: TCCGTGCACTTACGGTTAGTTTT<br>P: CCAGAGCCATTCAGT	C043R053	2.16
Ldhb	L-lactate dehydrogenase B-A chain-like	Host gene	F: GTCACTGCTCCCATTTTACACTCTAG<br>R: CCCAAACTCCCTCCCAGATAAC<br>P: CTGTTCTTAGCTTCCC	C161R138	N/A*
Map3k14	Mitogen-activated protein kinase 14-like	Host gene	F: GCTCCCTGGGTTCATGGAT<br>R: GCCTCCCTTCAGCAGAGACA<br>P: CCAGCAATAGCTTATG	C014R149, C060R057	2.02
MHC1	Major histone compatibility complex 1	Host gene	F: GCGACAGGTTTCTACCCCAGT<br>R: TGTCAGGTGGGAGCTTTTCTG<br>P: TGGTGTCCTGGCAGAAAGACGG	CX141267	2.19
MHCII-B	Major histone compatibility complex class II	Host gene	F: TGCCATGCTGATGTGCAG<br>R: GTCCCTCAGCCAGGTCACT<br>P: CGCCTATGACTTCTACCCCAAACAAAT	AF115533	1.99
MMP13	Matrix metalloproteinase 13	Host gene	F: GCCAGCGGAGCAGGAA<br>R: AGTCACCTGGAGGCCAAAGA<br>P: TCAGCGAGATGCAAAG	NM_001140524	1.99
MMP25	Matrix metalloproteinase 25	Host gene	F: TGCAGTCTTTTCCCCTTGGAT<br>R: TCCACATGTACCCACACCTACAC<br>P: AGGATTGGCTGGAAGGT	CB516773	2.03
MPDU1	Mannose-P-Dolichol Utilization	Host gene	F: TGCTTGACCCCTTGATTATAGCTA	C007R028	1.95

Symbol	Infectious agent/<br>Host gene name	Assay Class	Forward Primer Sequence (5'-3'),<br>Reverse Primer Sequence (5'-3'),<br>Probe Sequence (FAM-5'-3'-MGB)	Accession number	Amplification Factor
	Defect 1		R: GACCATAATCTAGAATGAAAACGCATT<br>P: CTTCCTGGTTGTGTTCTG		
Mx_onts	Antiviral protein	Host gene	F: CCACTTGCCAGAGCATGGT<br>R: CGTAACTGCCCAGAGTGCAAT<br>P: ATTCCCATGGTGATCCGCTACCTGG		2.01
NFX	Zinc finger NFX1-type	Host gene	F: CCACTTGCCAGAGCATGGT<br>R: CGTAACTGCCCAGAGTGCAAT<br>P: TGCTCCACCGATCG	FQ635861	1.96
NKA_a3	Na+/K+ ATPase subunit a3	Host gene	F: GGAGACCAGCAGAGGAACAG<br>R: CCCTACCAGCCCTCTGAGT<br>P: AAGACCCAGCCTGAAATG	CK170270	2.15
NKA_b1	Na+/K+ ATPase subunit b1	Host gene	F: CGTCAAGCTGAACAGGATCGT<br>R: CCTCAGGGATGCTTTCATTGGA<br>P: CCTTGGCCTGAAGTTG	CK886866	1.98
NKAa1-a	Na+/K+ ATPase subunit α-1a	Host gene	F: TGGAATCAAGGTTATCATGGTCACT<br>R: CCCACACCCTTGGCAATG<br>P: ATCATCCCATCACTGCGA	C217R121	2.06
NKAa1-b	Na+/K+ ATPase subunit α-1b	Host gene	F: GCCTGGTGAAGAATCTTGAAGCT<br>R: GAGTCAGGGTTCCGGTCTTG<br>P: CCTCCACCATTTGCTCA	C230R144	2.22*
NKAA1C	Na+/K+ ATPase subunit 1c	Host gene	F: AGGGAGACGTACTACTAGAAAGCAT<br>R: CAGAACTTAAAATTCCGAGCAGCAA<br>P: ACAACCATGCAAGAACT	CK885259	2.02
park7	Protein deglycase DJ-1	Host gene	F: ACTGCAAGCAGCATGATCAACT<br>R: TTGGCCTGTGTATCATAATGAACA<br>P: CCCCACCTACTCAGC	C246R153	2.19
PCBL	Precerebellin	Host gene	F: TGGTGTTGCTTTGCTGTTGT<br>R: GCCACTTTTGGTTTGCTCTC<br>P: ATGGTTGAGACTCAGACGGAGAGTG	AF192969	2.01
PDIA4	Protein disulfide-isomerase A4	Host gene	F: TGAGGTGCAGGACTTTTTTAAGAA	C263R094	2.02

Symbol	Infectious agent/<br>Host gene name	Assay Class	Forward Primer Sequence (5'-3'),<br>Reverse Primer Sequence (5'-3'),<br>Probe Sequence (FAM-5'-3'-MGB)	Accession number	Amplification Factor
			R: TCGTTGCTCTGTTTCCTGTGA		
			P: ACATCCTGCCACTGGT		
PgK3	Phosphoglycerate kinase 3	Host gene	F: GGCAAAGTGCTCCCTAAGTTTC	100194765	2.09
			R: TAGAGAGCAGGGCTGGTGCTA		
			P: CACCCTGCGCTTGT		
PRAS	G-protein mRNA	Host gene	F: GCAGGATGAGCAGAGGAAGAA	CA059617	2.00
			R: GGCCTGGGCAATGTAACACT		
			P: CCCCCTAAAGATGCAG		
RPL31	60S Ribosomal protein L31	Host gene	F: GAGTACACGGTCAACATCCACAA	C209R008	2.06
			R: CGAGGTGCCCTCCTCTTAAA		
			P: CGCATACATGGCGTCT		
RPL6	Ribosomal protein L6	Host gene	F: CGCCACCACAACCAAGGT	CB495313	1.96
			R: TCCTCAGCCTCTTCTTCTTGAAG		
			P: AGATCCCCAAGACTCTGTCAGACGCCT		
RSAD	Radical S-adenosyl methionine domain-containing protein 2	Host gene	F: GCCATTGCTGACAATACTGACACT	209733083,	2.04
			R: GCCATTGCTGACAATACTGACACT	DY728694	
			P: GGGAAATTAGTCCAATACTGCAAAC		
SAA	Serum amyloid protein a	Host gene	F: GGGAGATGATTCAGGGTTCCA	X99387	2.01
			R: TTACGTCCCCAGTGGTTAGC		
			P: TCGAGGACACGAGGACTCAGCA		
SCG	secretogranin II [Ctenopharyngodon idella]	Host gene	F: GGATGTGAAGAATCCAACACTGAT	CA053613	1.96
			R: ACACCACTTCAAACTAGCCATACATT		
			P: 6FAM-CGGCTGTATGTGCACTG-MGBNFQ		
sepw1	Selenoprotein W	Host gene	F: TGAGGATGAATTCCCAGGTGAT	C035R144,	2.03
			R: AAACCACCCAGAGGTTGAAGGT	C258R108,	
			P: TTGAGATTACTGGTGAAAGC	C052R159,	
				C155R164,<br>C142R109	
SERPIN	Serpin H1 precursor	Host gene	F: GAGGTCAGCGACCCAAAGAC<br>R: GCCGTAGAGGCGGTTACTGAT	C236R132	2.02
			P: CGGAACGTCACATGGA		
SHOP21	Hyperosmotic protein 21	Host gene	F: GCGGTAGTGGAGTCAGTTGGA	CA054269	2.66*

Symbol	Infectious agent/ Host gene name	Assay Class	Forward Primer Sequence (5'-3'), Reverse Primer Sequence (5'-3'), Probe Sequence (FAM-5'-3'-MGB)	Accession number	Amplification Factor
			R: GCTGCTGACGTCTCACATCAC<br>P: CCTGTTGATGCTCAAGG		
SRK2	Tyrosine-protein kinase SKR2	Host gene	F: CCAACGAGAAGTTCACCATCAA<br>R: TCATGATCTCATACAGCAAGATTCC<br>P: TGTGACGTGTGGTCCT	209730585, CA349577	2.12
STAT1	Signal transducer and activator of transcription 1-alpha/beta	Host gene	F: TGTCACCGTCTCAGACAGATCTG<br>R: TGTTGGTCTCTGTAAGGCAACGT<br>P: AGTTGCTGAAAACCGG	CB517962	1.90
TCRa	T-cell receptor alpha	Host gene	F: ACAGCTTGCCTGGCTACAGA<br>R: TGTCCCCTTTCACTCTGGTG<br>P: CAGCGCACACAAGGCTAATTCG	AY552002	2.12
TCRb	T-cell receptor beta	Host gene	F: TCACCAGCAGACTGAGAGTCC<br>R: AAGCTGACAATGCAGGTGAATC<br>P: CCAATGAATGGCACAAACCAGAGAA	AF329700	2.11
TF	transferrin	Host gene	F: TTCACTGCTGGAAAATGTGG<br>R: GCTGCACTGAACTGCATCAT<br>P: TGGTCCCTGTCATGGTGGAGCA	D89083	2.13
TNF	Tumor necrosis factor	Host gene	F: CCCACCATACATTGAAGCAGATT<br>R: GGATTGTATTCACCCTCTAAATGGA<br>P: CCGGCAATGCAAAA	AJ277604	2.00
Tuba1a	Tubulin alpha-1A chain	Host gene	F: CTCTGCTGAGAAGGCCTACCAT<br>R: AGCAGGCGTTGGTGATGTC<br>P: AGCAGCTGTCTGTTGC	C036R054, C062R114, C075R011	1.92
UBE2Q2	Ubiquitin-conjugating enzyme E2 Q2-like	Host gene	F: GGCAGGACCACTTGAACGTAA<br>R: AGGCCTGCACTGAACCAGAT<br>P: TGCTCATTCGGGTGCG	C182R024, C089R055	2.06
VHSV-P10	VHSV-induced protein-10 mRNA	Host gene	F: GCAAACTGAGAAAACCATCAAGAA<br>R: CCGTCAGCTCCCTCTGCAT<br>P: TGTGGAGAAGTTGCAGGC	CA040505	1.97
VHSVIP4	VHSV-inducible protein-4	Host gene	F: TGGCTTCCCACATTGCAA<br>R: CCTCCTCCCCCCTGCAT	GO053979	1.88

Symbol	Infectious agent/<br>Host gene name	Assay Class	Forward Primer Sequence (5'-3'),<br>Reverse Primer Sequence (5'-3'),<br>Probe Sequence (FAM-5'-3'-MGB)	Accession number	Amplification Factor
			P: AGATGGAGACAGGAATG		
ZAP7	Tyrosine-protein kinase ZAP-70	Host gene	F: TCACCTCCGGACCTTTCATT<br>R: CCATGTGGGAAGCCTTTTCTT<br>P: 6FAM-TCTTGTATGGTTTTCCTCC-MGBNFQ	CA052716	2.74*
78d16.1	S100 calcium binding protein	Reference Gene	F: GTCAAGACTGGAGGCTCAGAG<br>R: GATCAAGCCCCAGAAGTGTTTG<br>P: AAGGTGATTCCCTCGCCGTCCGA	CA056739	1.95
COIL-p84	Coiled-coil domain-containing protein 84	Reference Gene	F: GCTCATTTGAGGAGAAGGAGGATG<br>R: CTGGCGATGCTGTTCCTGAG<br>P: TTATCAAGCAGCAAGCC	CA053789	2.01
MrpL40	39S ribosomal protein L40	Reference Gene	F: CCCAGTATGAGGCACCTGAAGG<br>R: GTTAATGCTGCCACCCTCTCAC<br>P: ACAACAACATCACCA	CK991258	1.98
ae_hyd	Aeromonas hydrophila	Infectious agent	F: ACCGCTGCTCATTACTCTGATG<br>R: CCAACCCAGACGGGAAGAA<br>P: TGATGGTGAGCTGGTTG	AY165026	1.03
ae_sal	*Aeromonas salmonicida*	Infectious agent	F: TAAAGCACTGTCTGTTACC<br>R: GCTACTTCACCCTGATTGG<br>P: ACATCAGCAGGCTTCAGAGTCACTG	M64655	0.95
c_b_cys	‘*Candidatus* Branchiomonas cysticola’	Infectious agent	F: AATACATCGGAACGTGTCTAGTG<br>R: GCCATCAGCCGCTCATGTG<br>P: CTCGGTCCCAGGCTTTCCTCTCCCA	JQ723599	0.95
fl_psy	*Flavobacterium psychrophilum*	Infectious agent	F: GATCCTTATTCTCACAGTACCGTCAA<br>R: TGTAAACTGCTTTTGCACAGGAA<br>P: AAACACTCGGTCGTGACC	MN049781	0.86
sch	*Candidatus* Syngnamydia salmonis	Infectious agent	F: GGGTAGCCCGATATCTTCAAAGT<br>R: CCCATGAGCCGCTCTCTCT<br>P: TCCTTCGGGACCTTAC	FJ897519	1.09
mo_vis	*Moritella viscosa*	Infectious agent	F: CGTTGCGAATGCAGAGGT<br>R: AGGCATTGCTTGCTGGTTA	EU332345	0.96

Symbol	Infectious agent/<br>Host gene name	Assay Class	Forward Primer Sequence (5'-3'),<br>Reverse Primer Sequence (5'-3'),<br>Probe Sequence (FAM-5'-3'-MGB)	Accession number	Amplification Factor
			P: TGCAGGCAAGCCAACTTCGACA		
pch_sal	*Piscichlamydia salmonis*	Infectious agent	F: TCACCCCCAGGCTGCTT<br>R: GAATTCCATTTCCCCCTCTTG<br>P: CAAAACTGCTAGACTAGAGT	EU326495	0.97
pisck_sal	*Piscirickettsia salmonis*	Infectious agent	F: TCTGGGAAGTGTGGCGATAGA<br>R: TCCCGACCTACTCTTGTTTCATC<br>P: TGATAGCCCCGTACACGAAACGGCATA	U36943	0.93
re_sal	*Renibacterium salmoninarum*	Infectious agent	F: CAACAGGGTGGTTATTCTGCTTTC<br>R: CTATAAGAGCCACCAGCTGCAA<br>P: CTCCAGCGCCGCAGGAGGAC	AF123890	0.96
rlo	Rickettsia-like organism	Infectious agent	F: GGCTCAACCCAAGAACTGCTT<br>R: GTGCAACAGCGTCAGTGACT<br>P: CCCAGATAACCGCCTTCGCCTCCG	EU555284	0.95
te_mar	*Tenacibaculum maritimum*	Infectious agent	F: TGCCTTCTACAGAGGGATAGCC<br>R: CTATCGTTGCCATGGTAAGCCG<br>P: CACTTTGGAATGGCATCG	LC475109	1.03
vi_ang	*Vibrio anguillarum*	Infectious agent	F: CCGTCATGCTATCTAGAGATGTATTTGA<br>R: CCATACGCAGCCAAAAATCA<br>P: TCATTTCGACGAGCGTCTTGTTCAGC	L08012	0.95
vi_sal	*Vibrio salmonicida*	Infectious agent	F: GTGTGATGACCGTTCCATATTT<br>R: GCTATTGTCATCACTCTGTTTCTT<br>P: TCGCTTCATGTTGTGTAATTAGGAGCGA	AF452135	0.96
ye_ruc_glnA	*Yersinia ruckeri*	Infectious agent	F: TCCAGCACCAAATACGAAGG<br>R: ACATGGCAGAACGCAGAT<br>P: AAGGCGGTTACTTCCCGGTTCCC	FJ518778	1.04
de_sal	*Dermocystidium salmonis*	Infectious agent	F: CAGCCAATCCTTTCGCTTCT<br>R: GACGGACGCACACCACAGT<br>P: AAGCGGCGTGTGCC	U21337	1.01
ic_hof	*Ichthyophonus hoferi*	Infectious agent	F: GTCTGTACTGGTACGGCAGTTTC<br>R: TCCCGAACTCAGTAGACACTCAA	AF467793	0.93

Symbol	Infectious agent/ Host gene name	Assay Class	Forward Primer Sequence (5'-3'), Reverse Primer Sequence (5'-3'), Probe Sequence (FAM-5'-3'-MGB)	Accession number	Amplification Factor
			P: TAAGAGCACCCACTGCCTTCGAGAAGA		
sp_des	*Sphaerothecum destructuens*	Infectious agent	F: GGGTATCCTTCCTCTCGAAATTG	AY267346	0.99
			R: CCCAAACTCGACGCACACT		
			P: CGTGTGCGCTTAAT		
fa_mar	*Facilispora margolisi*	Infectious agent	F: AGGAAGGAGCACGCAAGAAC	HM800849	0.99
			R: CGCGTGCAGCCCAGTAC		
			P: TCAGTGATGCCCTCAGA		
lo_sal	*Loma salmonae*	Infectious agent	F: GGAGTCGCAGCGAAGATAGC	HM626243	1.04
			R: CTTTTCCTCCCTTTACTCATATGCTT		
			P: TGCCTGAAATCACGAGAGTGAGACTACCC		
nu_sal	*Nucleospora salmonis*	Infectious agent	F: GCCGCAGATCATTACTAAAAACCT	EF216905	0.94
			R: CGATCGCCGCATCTAAACA		
			P: CCCCGCGCATCCAGAAATACGC		
pa_ther	*Paranucleospora theridion*	Infectious agent	F: CGGACAGGGAGCATGGTATAG	FJ59481	0.93
			R: GGTCCAGGTTGGGTCTTGAG		
			P: TTGGCGAAGAATGAAA		
ce_sha	*Ceratonova shasta*	Infectious agent	F: CCAGCTTGAGATTAGCTCGGTAA	AF001579	0.93
			R: CCCCGGAACCCGAAAG		
			P: CGAGCCAAGTTGGTCTCTCCGTGAAAAC		
ku_thy	*Kudoa thyrsites*	Infectious agent	F: TGGCGGCCAAATCTAGGTT	AF031412	1.02
			R: GACCGCACACAAGAAGTTAATCC		
			P: TATCGCGAGAGCCGC		
my_arc	*Myxobolus arcticus*	Infectious agent	F: TGGTAGATACTGAATATCCGGGTTT<br>R: AACTGCGCGGTCAAAGTTG	HQ113227	0.96
			P: CGTTGATTGTGAGGTTGG		
my_ins	*Myxobolus insidiosus*	Infectious agent	F: CCAATTTGGGAGCGTCAAA	EU346375	0.95
			R: CGATCGGCAAAGTTATCTAGATTCA		
			P: CTCTCAAGGCATTTAT		
pa_kab	*Parvicapsula kabatai*	Infectious	F: CGACCATCTGCACGGTACTG	DQ515821	1.02

Symbol	Infectious agent/ Host gene name	Assay Class	Forward Primer Sequence (5'-3'), Reverse Primer Sequence (5'-3'), Probe Sequence (FAM-5'-3'-MGB)	Accession number	Amplification Factor
		agent	R: ACACCACAACTCTGCCTTCCA<br>P: CTTCGGGTAGGTCCGG		
pa_min	*Parvicapsula minibicornis*	Infectious agent	F: AATAGTTGTTTGTCGTGCACTCTGT<br>R: CCGATAGGCTATCCAGTACCTAGTAAG<br>P: TGTCCACCTAGTAAGGC	AF201375	0.95
pa_pse	*Parvicapsula pseudobranchicola*	Infectious agent	F: CAGCTCCAGTAGTGTATTTCA<br>R: TTGAGCACTCTGCTTTATTCAA<br>P: CGTATTGCTGTCTTTGACATGCAGT	AY308481	0.91
te_bry	*Tetracapsuloides bryosalmonae*	Infectious agent	F: GCGAGATTTGTTGCATTTAAAAAG<br>R: GCACATGCAGTGTCCAATCG<br>P: CAAAATTGTGGAACCGTCCGACTACGA	AF190669	0.98
gy_sal	*Gyrodactylus salaris*	Infectious agent	F: CGATCGTCACTCGGAATCG<br>R: GGTGGCGCACCTATTCTACA<br>P: TCTTATTAACCAGTTCTGC		0.95
na_sal	*Nanophyetus salmincola*	Infectious agent	F: CGATCTGCATTTGGTTCTGTAACA<br>R: CCAACGCCACAATGATAGCTATAC<br>P: TGAGGCGTGTTTTATG	AY269674	0.95
cr_sal	*Cryptobia salmositica*	Infectious agent	F: TCAGTGCCTTTCAGGACATC<br>R: GAGGCATCCACTCCAATAGAC<br>P: AGGAGGACATGGCAGCCTTTGTAT	AY713477	0.96
ic_mul	*Ichthyophthirius multifiliis*	Infectious agent	F: AAATGGGCATACGTTTGCAAA<br>R: AACCTGCCTGAAACACTCTAATTTTT<br>P: ACTCGGCCTTCACTGGTTCGACTTGG	IMU17354	1.00
ne_per	*Neoparamoeba perurans*	Infectious agent	F: GTTCTTTCGGGAGCTGGGAG<br>R: GAACTATCGCCGGCACAAAAG<br>P: CAATGCCATTCTTTTCGGA	EF216905	1.01
sp_sal	*Spironucleus salmonicida*	Infectious agent	F: GCAGCCGCGGTAATTCC<br>R: CGAACTTTTTAACTGCAGCAACA<br>P: ACACGGAGAGTATTCT	AY677182	0.98
ihnv	Infectious hematopoietic necrosis virus	Infectious agent	F: AGAGCCAAGGCACTGTGCG<br>R: TTCTTTGCGGCTTGGTTGA	NC_001652	0.98

Symbol	Infectious agent/ Host gene name	Assay Class	Forward Primer Sequence (5'-3'), Reverse Primer Sequence (5'-3'), Probe Sequence (FAM-5'-3'-MGB)	Accession number	Amplification Factor
			P: TGAGACTGAGCGGGACA		
ipnv	Infectious pancreatic necrosis virus	Infectious agent	F: GCAACTTACTTGAGATCCATTATGCT<br>R: GAGACCTCTAAGTTGTATGACGAGGTCTCT<br>P: CGAGAATGGGCCAGCAAGCA		0.86
isav7	Infectious salmon anemia virus	Infectious agent	F: CAGGGTTGTATCCATGGTTGAAATG<br>R: GTCCAGCCCTAAGCTCAACTC<br>P: CTCTCTCATTGTGATCCC	EU118822	0.89
isav8	Infectious salmon anemia virus	Infectious agent	F: TGGGCAATGGTGTATGGTATGA<br>R: GAAGTCGATGAACTGCAGCGA<br>P: CAGGATGCAGATGTATGC	EU118822	1.00
pspv	Pacific salmon parvovirus	Infectious agent	F: CCCTCAGGCTCCGATTTTTAT<br>R: CGAAGACAACATGGAGGTGACA<br>P: CAATTGGAGGCAACTGTA		0.93
pmcv	Piscine myocarditis virus	Infectious agent	F: AGGGAACAGGAGGAAGCAGAA<br>R: CGTAATCCGACATCATTTTGTGA<br>P: TGGTGGAGCGTTCAA	HQ401057	1.00
prv	Piscine orthoreovirus	Infectious agent	F: TGCTAACACTCCAGGAGTCATTG<br>R: TGAATCCGCTGCAGATGAGTA<br>P: CGCCGGTAGCTCT	QIA46570	1.01
sav	Salmon alphavirus 1, 2, and 3	Infectious agent	F: CCGGCCCTGAACCAGTT<br>R: GTAGCCAAGTGGGAGAAAGCT<br>P: TCGAAGTGGTGGCCAG	AY604235	0.95
omv	Salmonid herpesvirus / Oncorhynchus masou herpes virus	Infectious agent	F: GCCTGGACCACAATCTCAATG<br>R: CGAGACAGTGTGGCAAGACAAC<br>P: CCAACAGGATGGTCATTA		0.98
ver	Viral encephalopathy and retinopathy virus	Infectious agent	F: TTCCAGCGATACGCTGTTGA<br>R: CACCGCCCGTGTTTGC<br>P: AAATTCAGCCAATGTGCCCC	AJ245641	0.94
ven	Viral erythrocytic necrosis virus	Infectious agent	F: CGTAGGGCCCCAATAGTTTCT<br>R: GGAGGAAATGCAGACAAGATTTG		0.93

Symbol	Infectious agent/<br>Host gene name	Assay Class	Forward Primer Sequence (5'-3'),<br>Reverse Primer Sequence (5'-3'),<br>Probe Sequence (FAM-5'-3'-MGB)	Accession number	Amplification Factor
			P: TCTTGCCGTTATTTCCAGCACCCG		
vhsv	Virus Viral hemorrhagic septicemia virus	Infectious agent	F: AAACTCGCAGGATGTGTGCGTCC<br>R: TCTGCGATCTCAGTCAGGATGAA<br>P: TAGAGGGCCTTGGTGATCTTCTG	Z93412	0.95

29

Table S3: Histopathology scoring across all tissues of Chinook salmon *(Oncorhynchus tshawytscha)* examined.

**Present Agent**	**Fish No.**	**Load (RNA copy number per µg of nucleic acids)**	**Used in ISH?**	Heart		Liver			Spleen				Kidney					Pancreas	GIT	Brain					Gills			Muscle
				Pericarditis	Endo/myocarditis	Congestion	Necrosis	Hepatitis	Congestion	Ellipsoids necrosis	Inflammation of the white pulp	Capsule proliferation	Nephritis	Nephrosis	Interstitial hyperplasia	Necrosis	Glomerulitis	Pancreatitis/ necrosis	Enteritis	Encephalitis	Malacia	Gliosis	Congestion	Microsporidian infection	Branchitis	Congestion	Epithelial proliferation	Myositis
Ca. B. cysticola	B6930	134918.18	yes	0	0	0	0	0	0	0	0	0	0	0	0	0	0	0	0	/	/	/	/	/	0	0	0	0
Ca. B. cysticola	B7122	512560.67	yes	0	0	0	0	0	0	1	2	0	0	0	1	0	0	0	0	/	/	/	/	/	0	0	0	0
Ca. B. cysticola	B6935	512560.67	yes	0	0	0	0	0	0	0	1	0	0	0	0	0	0	0	0	0	0	0	0	0	1	0	0	0
Ca. B. cysticola	D5396	360963.25	yes	0	0	0	0	0	0	0	0	0	0	0	2	0	0	0	0	0	0	0	0	0	1	0	0	0
Ca. B. cysticola	B6968	123607.01	yes	0	0	0	0	0	0	0	1	0	0	0	0	0	0	0	0	/	/	/	/	/	0	0	0	0
Ca. B. cysticola	B5102	80718.31	yes	0	0	0	0	0	0	0	0	0	0	0	1	0	0	0	0	/	/	/	/	/	0	0	0	/
Ca. B. cysticola	B5099	36973.24	yes	0	0	0	0	0	0	1	1	0	0	/	1	1	/	/	/	0	0	0	0	0	0	0	0	0
I. hoferi	B7134	183611.13	yes	2	1	0	0	0	0	0	2	0	0	0	2	0	0	0	0	/	/	/	/	/	0	0	0	0
I. hoferi	D3175	229153.09	yes	1	2	0	1	0	0	1	1	0	1	0	1	1	0	0	0	/	/	/	/	/	1	0	0	1
I. hoferi	B7066	107315.09	yes	0	0	0	0	0	0	0	1	0	0	0	0	1	0	0	0	/	/	/	/	/	0	0	0	0
I. hoferi	D4090	23407.79	yes	0	1	0	1	1	0	0	0	0	0	0	0	0	0	0	0	0	0	0	0	0	0	0	0	1
I. hoferi	B2184	681.55	yes	0	0	0	0	1	0	0	1	0	0	0	2	0	0	0	0	0	0	0	0	0	0	0	0	0
C. shasta	B5041	54837.28	yes	0	0	0	0	0	0	0	0	1	0	0	1	0	0	0	0	0	0	0	0	0	0	0	0	0
C. shasta	B5061	2116.98	no	0	0	0	0	1	0	0	1	0	2	0	0	0	2	0	0	0	0	0	0	0	0	0	0	0
C. shasta	B5066	2491.78	yes	0	0	0	0	0	0	0	0	2	2	1	1	0	0	2	2	0	0	0	0	0	1	0	1	0
C. shasta	B5077	2420.04	no	0	0	0	0	0	0	0	1	0	0	0	1	0	1	0	0	0	0	0	0	0	0	0	0	0
C. shasta	B5079	19283.60	yes	0	0	0	0	0	0	0	0	2	0	0	1	0	0	0	0	0	0	0	0	0	1	0	0	0
C. shasta	B5089	1957.00	yes	0	0	0	0	0	0	0	0	0	2	0	0	0	2	0	0	0	0	0	0	0	1	0	0	0
P. minibicornis	B5083	77897.61	yes	0	0	0	0	0	0	0	0	0	2	0	0	0	2	0	0	0	0	0	0	0	0	0	0	0
P. minibicornis	B5101	78168.90	yes	0	0	0	0	0	0	0	1	0	0	0	1	0	1	0	0	1	0	0	0	0	1	0	0	0
P. minibicornis	B7057	2064273.21	yes	0	0	0	0	1	0	0	1	0	0	0	0	0	0	0	0	0	0	0	0	0	0	0	0	0
P. minibicornis	B7091	136990.59	no	0	0	0	0	0	0	0	1	0	0	0	1	0	0	0	0	0	0	0	0	0	0	0	0	0

P. minibicornis	D5322	260090.25	no	0	0	0	0	0	0	0	0	0	0	0	0	0	0	0	0	0	0	0	0	0	0	0	0	0
P. minibicornis	D5326	161347.76	no	0	0	0	0	0	0	0	0	0	0	0	0	0	0	0	0	0	0	0	0	0	0	0	0	0
P. minibicornis	D4229	343318.54	no	0	0	0	0	0	0	0	0	0	0	0	0	0	0	0	0	0	0	0	0	0	0	0	0	0
P. minibicornis	D4235	1067319.50	no	0	0	0	0	0	0	0	0	0	0	0	1	0	0	0	0	0	0	0	0	0	0	0	0	0
P. minibicornis	D4236	466127.82	no	0	0	0	0	1	0	0	0	0	0	0	0	0	0	0	0	0	0	0	0	0	0	0	0	0
P. minibicornis	D4240	409888.39	no	0	0	0	0	0	0	0	0	0	0	0	0	0	0	0	0	0	0	0	0	0	0	0	0	0
P. minibicornis	D4274	413485.35	no	0	0	0	0	0	0	0	0	0	0	0	0	0	0	0	0	0	0	0	0	0	0	0	0	0
P. minibicornis	D4277	442712.75	no	0	0	0	0	0	0	0	0	0	0	0	0	0	0	0	0	0	0	0	0	0	0	0	0	0
P. minibicornis	D4278	78507.72	no	0	0	0	0	0	0	0	0	0	0	0	0	0	0	0	0	0	0	0	0	0	0	0	0	0
P. minibicornis	B2163	1447.83	no	0	0	0	0	0	0	0	2	0	0	0	2	0	0	0	0	0	0	0	0	0	0	0	0	0
P. theridion	B2208	5390.93	no	0	0	0	0	0	0	0	1	2	0	1	1	0	0	0	0	0	0	0	0	0	1	0	0	0
P. theridion	B5027	501.93	no	0	0	0	0	0	0	0	0	0	0	0	0	0	0	0	0	0	0	0	0	0	1	0	0	0
P. theridion	B5040	751.94	no	0	0	0	0	0	0	0	1	0	0	0	0	0	0	0	0	0	0	0	0	0	1	0	0	0
P. theridion	D5396	324.27	no	0	0	0	0	0	0	0	1	0	0	0	1	0	0	0	0	1	0	0	0	0	1	0	0	0
PRV	B2157	1405.63	yes	0	0	0	0	0	0	0	0	0	0	0	1	0	0	0	0	0	0	0	0	0	0	0	0	0
PRV	B2159	654.94	yes	0	1	0	0	0	0	0	0	0	0	1	1	0	0	0	0	0	0	0	0	0	0	0	0	0
PRV	B2161	7535.33	yes	0	0	0	0	0	0	0	0	0	0	0	0	0	0	0	0	0	0	0	0	0	0	0	0	0
PRV	B2166	4245.96	yes	0	0	0	0	0	0	0	0	0	0	0	0	0	0	0	0	0	0	0	0	0	0	0	0	0
PRV	B2170	46.49	no	0	0	0	0	0	0	0	1	0	0	0	1	0	0	0	0	0	0	0	0	0	0	0	0	0
PRV	B2176	1024.36	yes	0	0	0	0	0	0	0	1	0	0	0	1	0	0	0	0	0	0	0	0	0	2	0	0	0
PRV	B2211	1241.60	yes	0	0	0	0	0	0	0	0	1	0	0	1	0	0	0	0	0	0	0	0	0	1	0	0	0
PRV	D5324	31926.07	yes	0	0	0	0	0	0	0	0	0	0	1	0	0	0	0	0	0	0	0	0	0	0	0	0	0

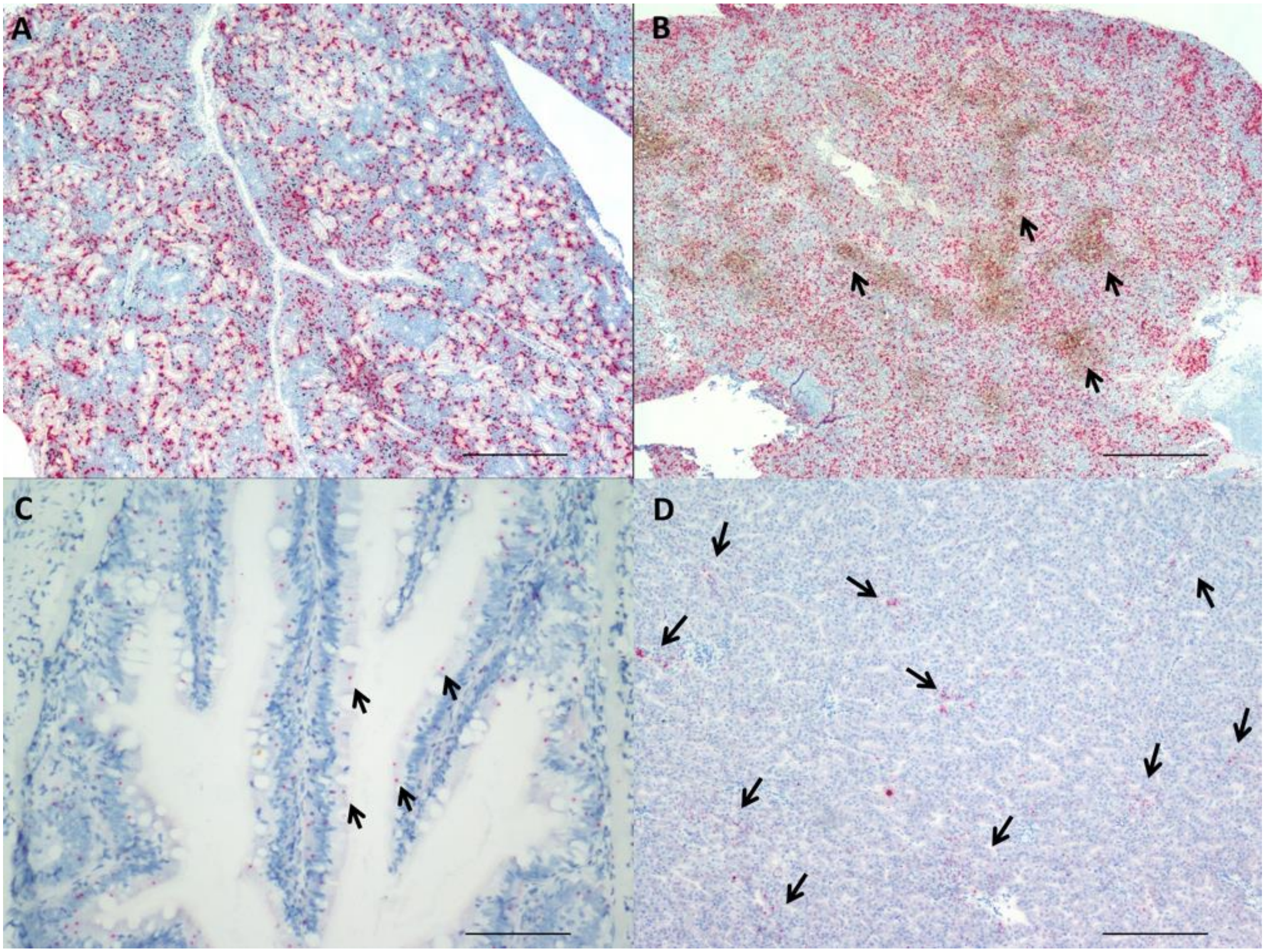

Figure S1: Detection of Piscine orthoreovirus (PRV) in the tissues (Fish B2159) by in-situ hybridization (ISH staining. Kidney (A) and spleen (B) were heavily infected with PRV (marked as red staining and red dots), which was observed in the vast majority of macrophagic cells and red blood cells; Arrows (in B) indicate the presence of hemosiderin in the spleen. PRV was also detected in the enterocytes in the intestine (C) and in several hepatocytes in the liver (D).

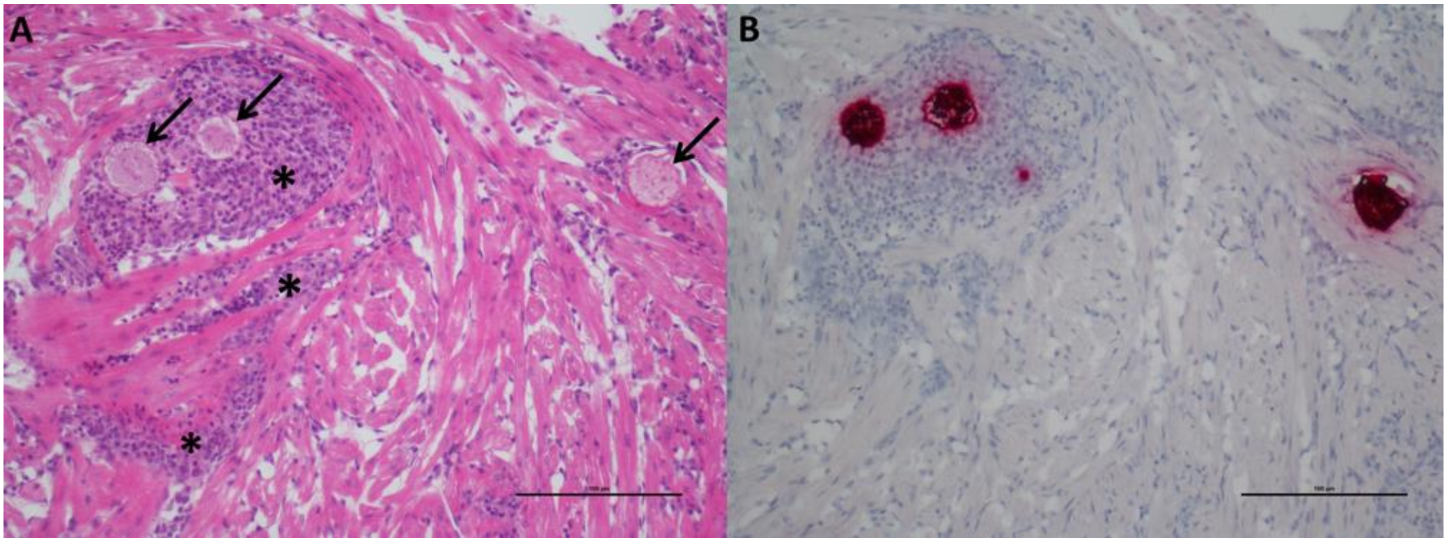

Figure S2: Fungal cysts in the endocardium contained *Ichthyophonus hoferi*. A) Fungal cysts (arrows), surrounded by granulomatous reactions as revealed by H&E staining (asterisk, scale bar = 100µm; Fish D3175). B) The same field under ISH shows the presence of *I. hoferi* in cysts (in red, scale bar = 100µm).

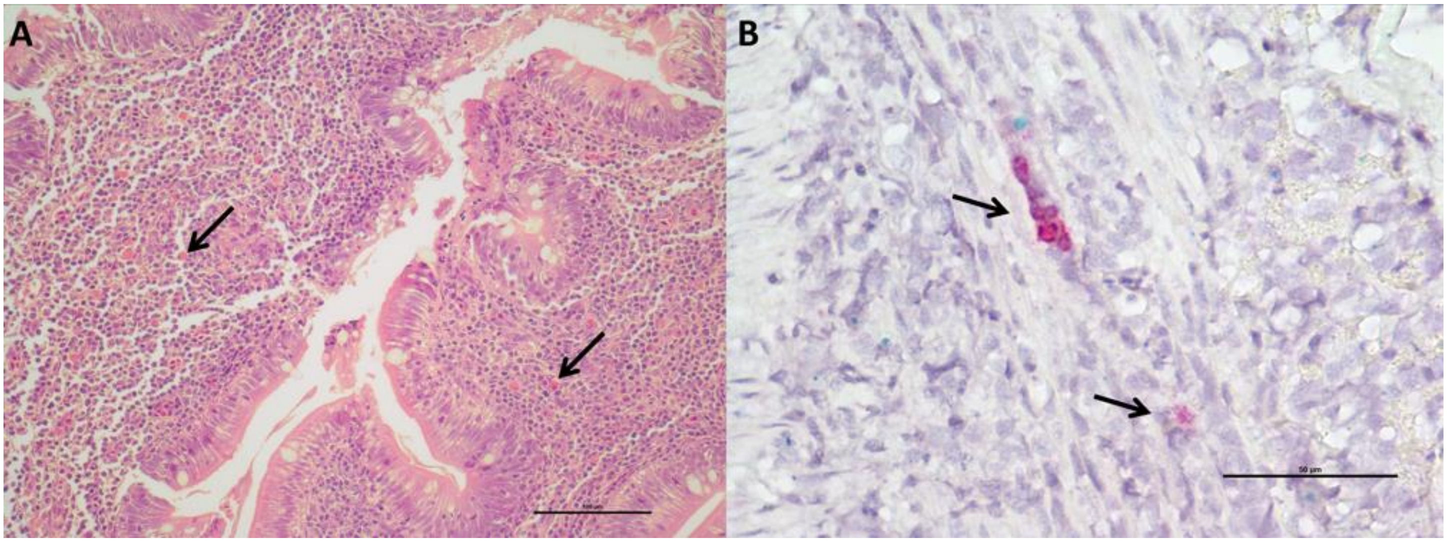

Figure S3: A fish infected with *Ceratonova shasta* exhibited chronic enteritis (Fish B5066). A) moderate chronic enteritis, affecting primarily the lamina propria in the intestine; several heterophilic granulocytes are observed (arrows; scale bar = 100µm). B) *Ceratonova shasta* detected through ISH in the lamina propria of the intestine, affected by chronic enteritis (red; scale bar 50µm). The blue spots in (B) are *Parvicapsula minibicornis.*

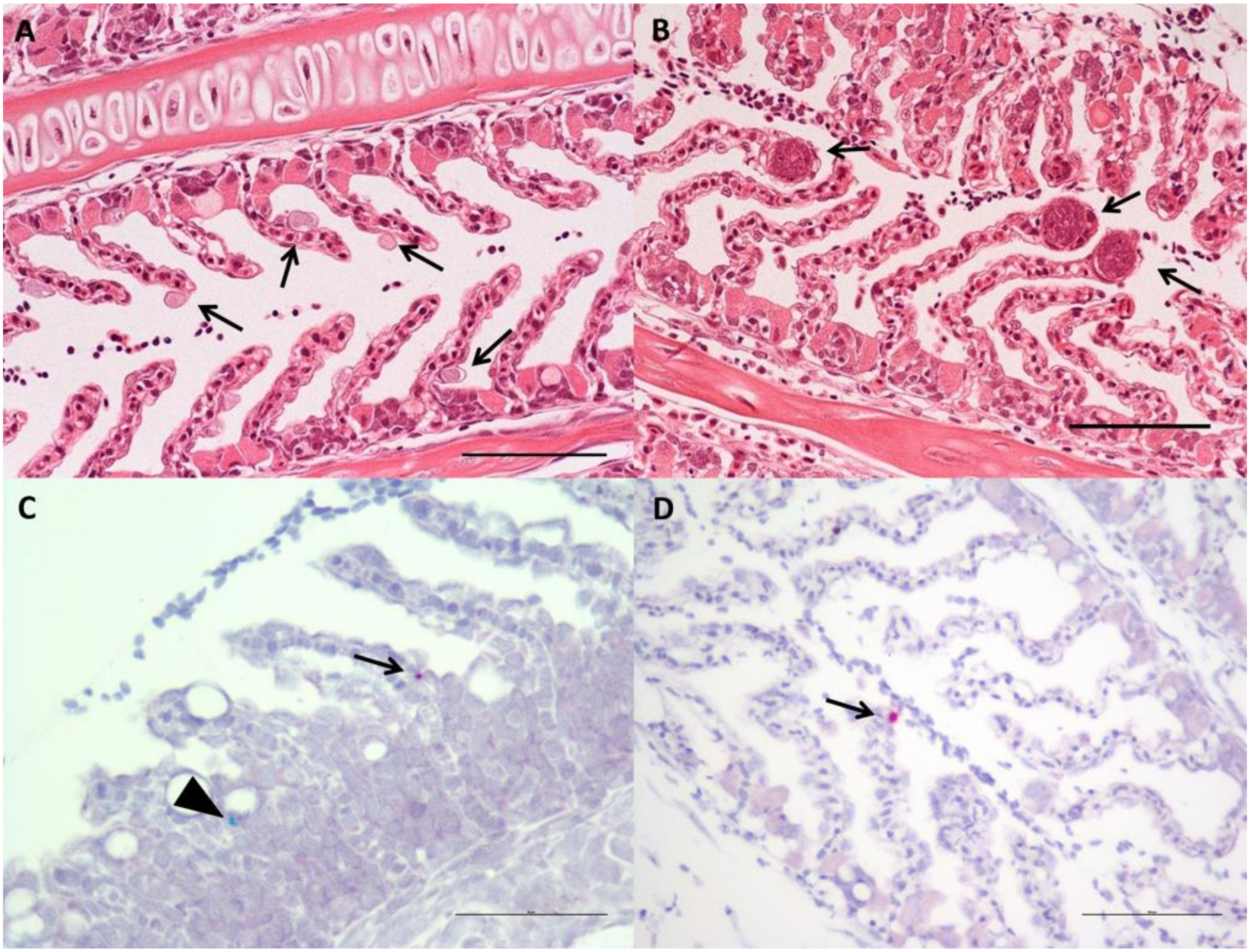

Figure S4: Lesions (H&E) and Ceratonova shasta detections (In-Situ Hybridization (ISH) staining) in gill tissues (Fish B5089); (A) chlamydia-like aggregates (epitheliocysts) in the lamellae in gills (scale bar 100µm;); (B) C. shasta infection: suspected pre-spore aggregates at tips of lamellae (scale bar 100µm); (C) C. shasta (red) detected through ISH in gills (scale bar 50µm); Parvicapsula minibicornis (blue) is marked by triangle head; (D) another C. shasta (red) detected through ISH in gills (scale bar 100µm);

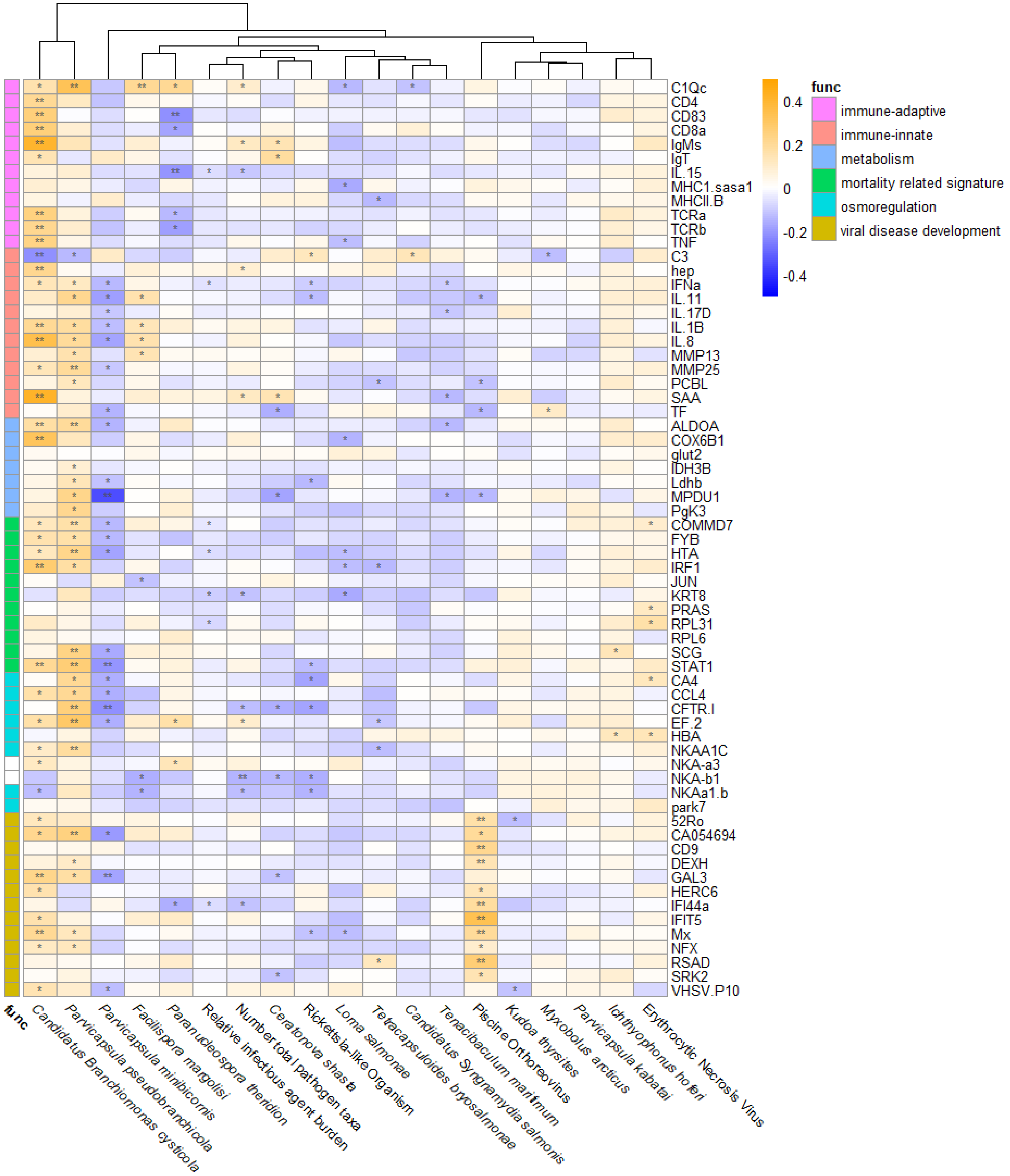

Figure S5: Heatmap of coefficients for each pathogen in models for individual genes in gill tissue. Cell values represent the change in units of standard deviation for the plasma or gene variable associated with a standard deviation increase in log pathogen load (from mixed tissue). Two asterisks in a cell indicate an FDR-adjusted p value < 0.05 and a single asterisk indicates a p value < 0.05 prior to FDR adjustment. Genes are grouped vertically by putative function.

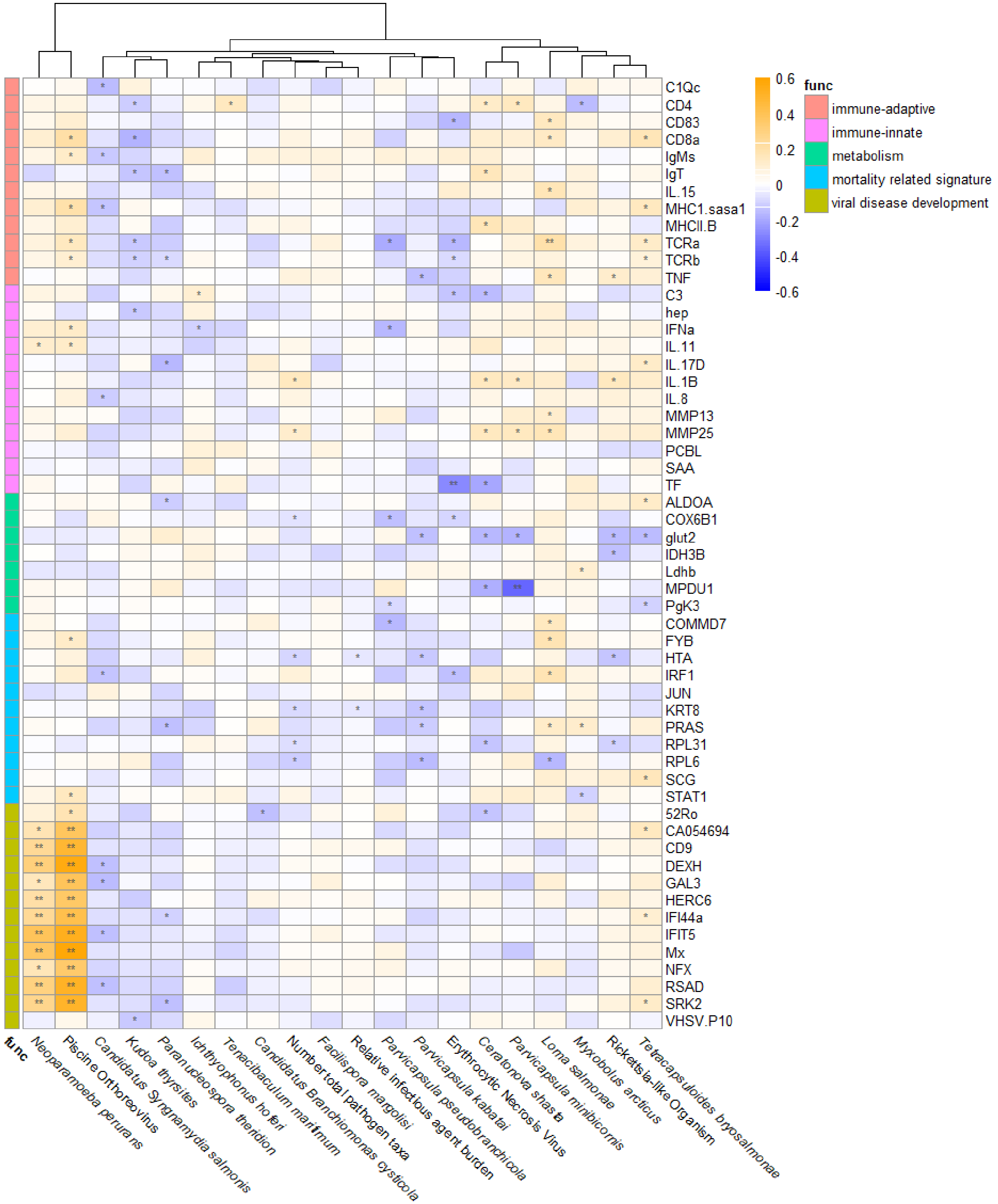

Figure S6: Heatmap of coefficients for each pathogen in models for individual genes in liver tissue. Cell values represent the change in units of standard deviation for the plasma or gene variable associated with a standard deviation increase in log pathogen load (from mixed tissue). Two asterisks in a cell indicate an FDR-adjusted p value < 0.05 and a single asterisk indicates a p value < 0.05 prior to FDR adjustment. Genes are grouped vertically by putative function.
